# Oligohistidine and targeting peptide functionalized TAT-NLS for enhancing cellular uptake and promoting angiogenesis in vivo

**DOI:** 10.1186/s12951-018-0358-x

**Published:** 2018-03-26

**Authors:** Qian Li, Xuefang Hao, Syed Saqib Ali Zaidi, Jintang Guo, Xiangkui Ren, Changcan Shi, Wencheng Zhang, Yakai Feng

**Affiliations:** 1 School of Chemical Engineering and Technology, Tianjin University, Tianjin, 300350 China; 2 Collaborative Innovation Center of Chemical Science and Chemical Engineering (Tianjin), Tianjin, 300350 China; 3 School of Ophthalmology, & Optometry, Eye Hospital, School of Biomedical Engineering, Wenzhou Medical University, Wenzhou, 325011 Zhejiang China; 4 Wenzhou Institute of Biomaterials and Engineering, CNITECH, CAS, Wenzhou, 325011 Zhejiang China; 5 Department of Physiology and Pathophysiology, Logistics University of Chinese People’s Armed Police Force, Tianjin, 300309 China; 6 Key Laboratory of Systems Bioengineering (Ministry of Education), Tianjin University, Tianjin, 300072 China

**Keywords:** Gene carrier, Peptide, Histidine, REDV, Targeting, HUVECs, pZNF580

## Abstract

**Background:**

Gene therapy has been developed and used in medical treatment for many years, especially for the enhancement of endothelialization and angiogenesis. But slow endosomal escape rate is still one of the major barriers to successful gene delivery. In order to evaluate whether introducing oligohistidine (H_n_) sequence into gene carriers can promote endosomal escape and gene transfection or not, we designed and synthesized Arg-Glu-Asp-Val (REDV) peptide functionalized TAT-NLS-H_n_ (TAT: typical cell-penetrating peptide, NLS: nuclear localization signals, H_n_: oligohistidine sequence, n: 4, 8 and 12) peptides with different H_n_ sequence lengths. pEGFP-ZNF580 (pZNF580) was condensed by these peptides to form gene complexes, which were used to transfect human umbilical vein endothelial cells (HUVECs).

**Results:**

MTT assay showed that the gene complexes exhibited low cytotoxicity for HUVECs. The results of cellular uptake and co-localization ratio demonstrated that the gene complexes prepared from TAT-NLS-H_n_ with long H_n_ sequence (n = 12) benefited for high internalization efficiency of pZNF580. In addition, the results of western blot analysis and PCR assay of REDV-TAT-NLS-H_12_/pZNF580 complexes showed significantly enhanced gene expression at protein and mRNA level. Wound healing assay and transwell migration assay also confirmed the improved proliferation and migration ability of the transfected HUVECs by these complexes. Furthermore, the in vitro and in vivo angiogenesis assay illustrated that these complexes could promote the tube formation ability of HUVECs.

**Conclusion:**

The above results indicated that the delivery efficiency of pZNF580 and its expression could be enhanced by introducing H_n_ sequence into gene carriers. The H_n_ sequence in REDV-TAT-NLS-H_n_ is beneficial for high gene transfection. These REDV and H_n_ functionalized TAT-NLS peptides are promising gene carriers in gene therapy.

**Electronic supplementary material:**

The online version of this article (10.1186/s12951-018-0358-x) contains supplementary material, which is available to authorized users.

## Background

Nowadays, small-diameter artificial blood vessels (< 6 mm) have been used in clinical treatment of cardiovascular diseases, but their long-term patency rate remains low [1, 2]. To solve this problem, many surface modification strategies have been developed to enhance the hemocompatibility of artificial blood vascular materials, such as grafting zwitterionic polynorbornene, phosphorylcholine, heparin, gelatin or silk fibroin to avoid platelet adhesion and aggregation [3–7].

The re-endothelialization of small-diameter artificial blood vessels is beneficial for high long-term patency. Surface modification with bioactive peptides, such as Arg-Gly-Asp (RGD) [8], Cys-Ala-Gly (CAG) [9] and Arg-Glu-Asp-Val (REDV), can promote the attachment to endothelial cells (ECs). Among these peptides, REDV peptide can be specially recognized by α4β1 integrin, which is enriched in ECs but lacked in smooth muscle cells (SMCs) [10, 11]. Ji et al. proved that the combination of rapamycin-loaded polymer base layer and REDV peptide tethered top layer could promote the competitive adhesion of human umbilical vein endothelial cells (HUVECs) over human aortic smooth muscle cells, and enhanced in situ endothelialization [12]. Recently, our studies demonstrated that REDV-modified gene carriers could specially recognize ECs and selectively enhance transfection efficiency by transferring DNA into ECs so as to promote their proliferation and migration as well as vascularization in vitro and in vivo [13–16].

Gene therapy has been considered as an effective method for EC migration and proliferation, which benefits for rapid endothelialization and neovascularization [17]. For successful gene therapy, the major key task is to develop effective gene carriers. Compared with viral vectors, the synthesized gene carriers have gained much attention owing to their high safety, low cost and convenient preparation [18–20]. However, there still exist some biological barriers in gene delivery [21], such as low biocompatibility, DNA packaging ability, cellular uptake, endosomal escape [22] and DNA release [23].

Low cellular uptake is a major barrier in gene delivery because cell membrane acts as a significant physical obstacle for gene carriers. In recent years, gene delivery systems based on cationic peptides have been widely studied owing to their high biocompatibility and specific functions [24]. Compared with polymeric gene carriers, these peptide carriers exhibit nearly no cytotoxicity for cells. Cell-penetrating peptides (CPPs), a series of short arginine-rich cationic peptides, have the specific ability to cross cell membrane. They serve as a flexible strategy for overcoming the first barrier and further improving the gene delivery effect [25–29]. Tyr-Gly-Arg-Lys-Lys-Arg-Arg-Gln-Arg–Arg-Arg (YGRKKRRQRRR, TAT peptide) is one of non-specific selectively CPPs [30], which was derived from human immunodeficiency viruses 1 (HIV-1) [31]. TAT has been proven to promote cellular uptake and gene delivery because of its high cell membrane penetration. Gao et al. modified the poly(N-isopropylacrylamide) (PNIPAM) microgel particles with TAT peptide to form PNIPAM-FL-TAT particle, which showed significantly high cellular internalization compared with the PNIPAM group [32].

Another key issue in gene delivery is whether therapeutic DNAs can effectively cross the nuclear pore complexes (NPCs) on the nuclear membrane into nucleus. Nuclear access is limited by small nuclear pore diameter (only 9 nm), so only small molecules can be directly transported into nucleus through NPCs [33, 34]. Several methods have been investigated to specially translocate DNAs into nucleus, for example, gene carriers containing nuclear localization signal (NLS) peptides [35, 36]. One of the most well-known NLS peptides is Pro-Lys-Lys–Lys-Arg-Lys-Val sequence (PKKKRKV), which is derived from the large T antigen of the SV40 virus and has been reported to improve the nuclear access and enhance gene delivery successfully. Zhang et al. used TAT-PKKKRKV peptide to carry VEGF_165_ plasmid to promote the expression of VEGF_165_ protein and angiogenesis [37]. TAT-NLS can transfer various cell styles without specificity and selectivity because the TAT peptide sequence possesses the ability to quickly enter into almost all live cells [38].

After cellular uptake, the gene complexes should rapidly escape from endosomal/lysosomal compartment [39, 40]. Many studies focused on the promotion of endosomal/lysosomal escape. Polycations, such as polyethyleneimine and polyamidoamine dendrimers, have the ability to destroy the endosomal membrane and escape from endosomes owing to the proton sponge effect. Polycations can catch large amount of protons and cause the influx of Cl^−^, leading to the disruption of endosome and release of DNAs into cytoplasm [41–44]. Moreover, histidine (H)-enriched gene carriers can also break the endosomal membrane due to the protonation of its imidazole ring. Imidazole ring facilitates gene delivery from endosome into cytosol because of its pH proton sponge effect [45, 46]. It exhibits hydrophobic under physiological condition (pH = 7.4) and becomes hydrophilic via protonating the unsaturated nitrogen in imidazole group in an acidic environment, such as endosome/lysosome [47, 48]. At low pH value (pH < 7.4), H-enriched gene carriers rupture the endosomal membrane and escape from endosomes [49]. Herein, in this paper, we aimed to design and prepare the EC targeting gene carriers based on CPPs, REDV and oligohistidine (H_n_, n = 4, 8 and 12) to overcome the multiple barriers in the process of gene delivery. These multifunctional carriers possessed EC targeting function, high internalization efficiency, enhanced endosome/lysosome escape capacity as well as nucleus translocation ability.

**Fig. 1:**
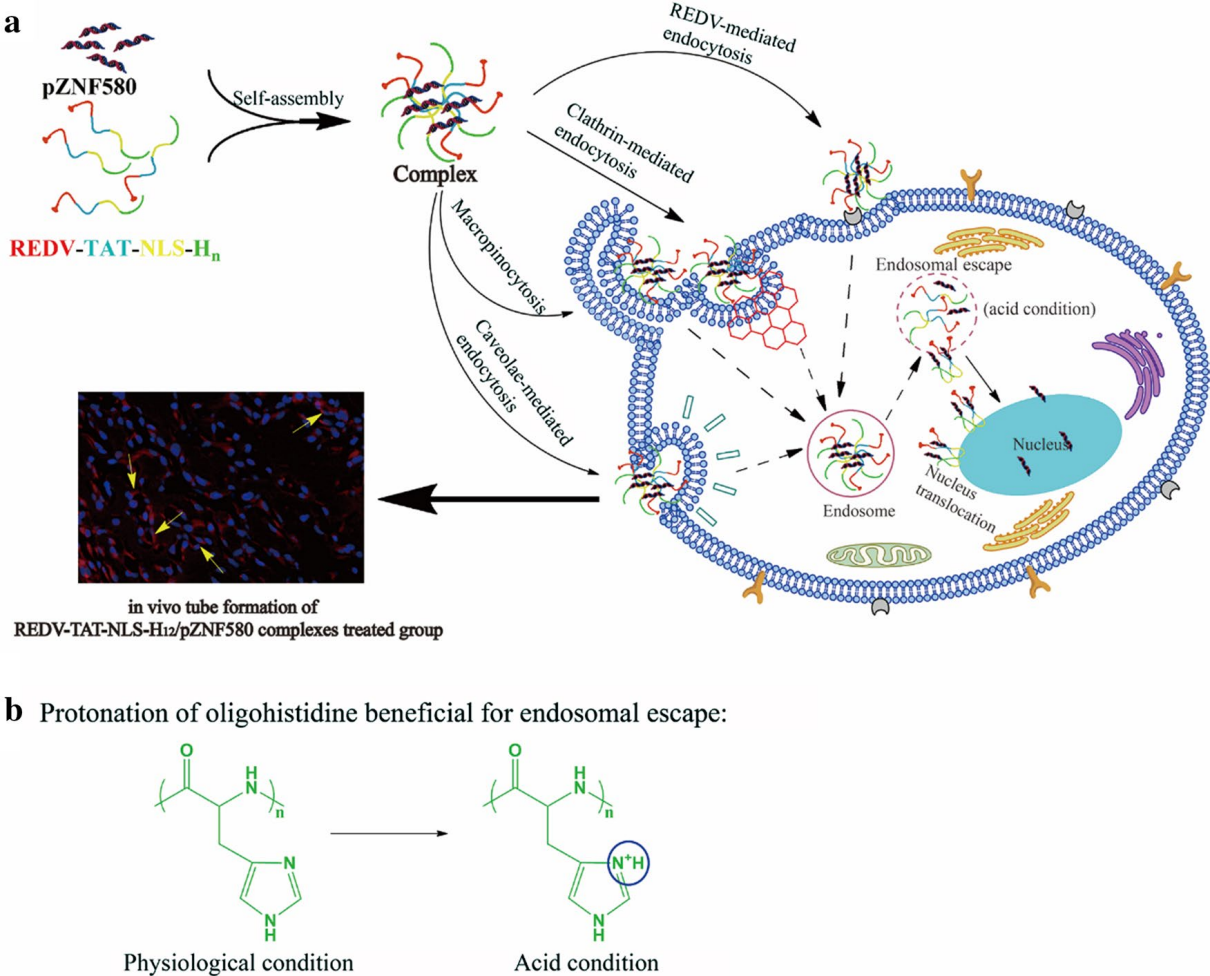
**a** Preparation process of REDV-TAT-NLS-H_n_/pZNF580 complexes and the illustration of gene delivery to ECs mediated by REDV-TAT-NLS-H_n_/pZNF580 complexes. **b** Protonation of oligohistidine beneficial for endosomal escape

In previous studies, enhanced green fluorescent protein (EGFP)-ZNF580 plasmid (abbreviated as pZNF580) was used to promote the proliferation and migration as well as angiogenesis ability of ECs. The expression of ZNF580 gene could promote vascular endothelial growth factor (VEGF) expression, which further enhanced the migration and proliferation of ECs [50–52]. In the present study, we synthesized the multifunctional peptide carriers with different length of oligohistidine sequence (REDV-TAT-NLS-H_4_, REDV-TAT-NLS-H_8_ and REDV-TAT-NLS-H_12_) in order to enhance their endosome/lysosome escape. These peptides could condense with pZNF580 gene to form gene complexes. The condensation ability was evaluated by agarose gel electrophoresis test. The cellular uptake processes of REDV-TAT-NLS-H_n_/pZNF580 (abbreviated as peptide/pZNF580) complexes, delivery of genes into ECs and angiogenesis were illustrated in Fig. 1a. Oligohistidine sequence had many imidazole groups that undergo protonation in endosome, which facilitated for endosome/lysosome escape and gene delivery (Fig. 1b). The cell viability of the peptide/pZNF580 complexes were determined by MTT test. Furthermore, the cellular uptake and intracellular trafficking of the peptide/pZNF580 complexes were measured quantitatively and qualitatively by flow cytometry and confocal laser scanning microscopy (CLSM). Western blot analysis and PCR assay were used to evaluate ZNF580 gene expression of the transfected cells by different peptide/pZNF580 complexes both at protein and mRNA level. The angiogenesis and tube formation ability of the transfected cells by peptide/pZNF580 complexes was evaluated in vitro and in vivo.

## Experimental section

### Materials

Peptides named REDV, REDV-YGRKKRRQRRR-PKKKRKV (abbreviated as REDV-TAT-NLS-H_0_), REDV-YGRKKRRQRRR-PKKKRKV-HHHH (abbreviated as REDV-TAT-NLS-H_4_), REDV-YGRKKRRQRRR-PKKKRKV-HHHHHHHH (abbreviated as REDV-TAT-NLS-H_8_) and REDV-YGRKKRRQRRR-PKKKRKV-HHHHHHHHHHHH (abbreviated as REDV-TAT-NLS-H_12_) were synthesized by GL Biochem. Ltd. (Shanghai, China). 3-(4,5-Dimethyl-2-thiazolyl)-2,5-diphenyl-2-H-tetrazolium bromide (MTT) was obtained from Ding Guo Chang Sheng Biotech. Co., Ltd. (Beijing, China). Dulbecco’s modified eagle medium (DMEM) and fetal bovine serum (FBS) were purchased from Invitrogen Corporation (Carlsbad, CA). Transwell chambers and Matrigel (Cat. Nos. 356234) was obtained from Corning Incorporated (New York, USA). TransScript First-Strand cDNA Synthesis SuperMix and TransStart™ Top Green qPCR SuperMix were purchased from Transgen Biotech Co., Ltd. (Beijing, China). BCA protein assay kit was obtained from Solarbio Science and Technology Co., Ltd. (Beijing, China). Rabbit anti-human ZNF580 polyclonal antibody, goat anti-rabbit IgG, amiloride hydrochloride, mouse anti-CD31 antibody and goat anti-mouse IgG H&L secondary antibody (Alexa Fluor^®^ 594) were purchased from Abcam Ltd. (Shanghai, China). Rabbit anti-beta-actin antibody was obtained from Beijing Biosynthesis Biotechnology Co., Ltd. (Beijing, China). Cy5 labeled oligonucleotide (Cy5-oligonucleotide) was purchased from Sangon Biotech Co., Ltd. (Shanghai, China). LysoTracker Green DND-26 and Hoechst 33342 were obtained from Shanghai Invitrogen Biotechnology Co., Ltd. (Shanghai, China). Chlorpromazine hydrochloride was purchased from Sigma-Aldrich (St. Louis, USA). Filipin III was purchased from Cayman Chemical (Michigan, USA). Human umbilical vein endothelial cells (HUVECs) were obtained from the Cell Bank of Typical Culture Collection of Chinese Academy of Sciences (Shanghai, China). The pZNF580-ZNF580 plasmid (pZNF580), smooth muscle cells (SMCs) and male mice were preserved by department of physiology and pathophysiology, Logistics University of Chinese People’s Armed Police Force.

### Preparation and characterization of complexes

#### Preparation of REDV-TAT-NLS-H_n_/pZNF580 complexes

The pZNF580 plasmid was diluted to 50 μg mL^−1^ with PBS (pH = 7.4) buffer. The REDV-TAT-NLS-H_n_/pZNF580 complexes were prepared by mixing REDV-TAT-NLS-H_n_ solution (0.5 mg mL^−1^) and plasmid solution with various weight ratios ranging from 0.5 to 5. The gene complex solutions were stirred for 30 min at room temperature and used for following experiments.

#### Particle size and zeta potential measurements

The average particle size and zeta potential of REDV-TAT-NLS-H_n_ and REDV-TAT-NLS-H_n_/pZNF580 complexes were measured using a Zetasizer Nano ZS (Malvern Instrument, Inc., Worcestershire, UK) with a constant angle of 173°.

#### Hemolysis assay

The hemocompatibility of peptide solutions were measured by hemolysis assay. Firstly, fresh whole blood was collected into heparin sodium vacuum blood collection tubes and centrifuged at 3000 rpm for 5 min. After washing, the red blood cells (RBCs) were suspended in 0.01 M PBS (pH = 7.4). Various REDV-TAT-NLS-H_n_ solutions (1000 μL) were added to the RBCs solution (25 μL) and incubated for 24 h. PBS (pH = 7.4) and purified water were used as negative and positive control, respectively. The absorbance of supernatant was measured at 540 nm. The hemolysis values of REDV-TAT-NLS-H_n_ solutions were calculated by the following equation:

$$ {\text{Hemolysis}}\;(\% ) = \frac{{{\text{OD}}_{\text{p}} - {\text{OD}}_{\text{nc}} }}{{{\text{OD}}_{\text{pc}} - {\text{OD}}_{\text{nc}} }} \times 100\% $$

where the OD_p_ is the absorbance of REDV-TAT-NLS-H_n_ solution samples, the OD_pc_ is the absorbance of positive control, and the OD_nc_ is the absorbance of negative control.

#### Agarose gel electrophoresis

The condensing ability of different pZNF580 complexes was evaluated by agarose gel electrophoresis assay. Briefly, the gene complexes were mixed with 6× loading buffer and electrophoresed on the 0.8% agarose gel containing 5 μL GoldView with 1× TAE buffer at 120 V for 25 min. Naked DNA was used as control. A UV illuminator was used to visualize the location of DNA bonds.

### Cell culture

Human umbilical vein endothelial cells (HUVECs) and human umbilical artery smooth muscle cells (HUASMCs) were, respectively plated in cell culture flasks and incubated with DMEM containing 10% FBS at 37 °C in humidified atmosphere with 5% CO_2_. The cells were cultured to reach 80–90% confluence before use.

### In vitro cytotoxicity

The cytotoxicity of REDV-TAT-NLS-H_n_ and REDV-TAT-NLS-H_n_/pZNF580 complexes for HUVECs was evaluated by MTT assay. Briefly, cells (1 × 10^4^ cells/well) were plated in 96-well plate and cultured in DMEM containing 10% FBS for 24 h to achieve 80% confluence. Then the medium was removed and serum-free medium was added to each well and cultured overnight. After that, various REDV-TAT-NLS-H_n_ solutions and pZNF580 complexes at various concentrations ranging from 5 to 120 μg mL^−1^ were added and incubated for 48 h. Then, MTT reagent (20 μL, 5 mg mL^−1^) was added to each well and cultured for another 4 h to form formazan crystals. Then, the medium was removed, followed by adding 150 μL DMSO to dissolve the formazan crystals. The optical density was measured at 490 nm using a microplate reader (BIO-RAD, iMark™, USA). The relative cell viability was calculated as: relative cell viability (%) = (OD490_(sample)_/OD490_(control)_) × 100%, where OD490_(sample)_ represents the absorbance value of experimental wells minus zero wells, and OD490_(control)_ represents the absorbance value of untreated cell wells minus zero wells).

### In vitro transfection

The transfection efficiency of REDV-TAT-NLS-H_n_/pZNF580 complexes was evaluated by HUVECs and HUASMCs. In brief, HUVECs and HUASMCs were seeded in 24-well plates at a density of 1 × 10^5^ cells per well and cultured with DMEM containing 10% FBS until 70–80% confluence. Before transfection, the cells were starved with serum-free medium for 12 h. REDV-TAT-NLS-H_n_/pZNF580 complexes were added into each well (3 μg pZNF580 per well). After 4 h incubation, the medium was replaced with fresh DMEM containing 10% FBS and the cells were cultured for another 24 h in CO_2_ incubator. To detect the expression of pZNF580, green fluorescent protein was observed via an inverted fluorescent microscope (Fluorescence OLYMPUS U-RFLT50, microscopy Olympus DP72) at 24 h point.

### Cell migration assay

Wound healing assay was performed to investigate the migration ability of transfected HUVECs by various REDV-TAT-NLS-H_n_/pZNF580 complexes. HUVECs were seeded in a 24-well plate and transfected with various REDV-TAT-NLS-H_n_/pZNF580 complexes, respectively. After a 24 h culture, a straight line was scratched across each well using a 200 μL micropipettor tip. The wells were washed twice with D-Hanks and cultured at 37 °C. The images of cell migration were obtained with an inverted microscope at 0, 6 and 12 h. The relative recovered area was calculated using Image J software with the following equation.

$$ {\text{Relative}}\;{\text{recovered}}\;{\text{area}}\;(\% ) = \frac{{{\text{Recovered}}\;{\text{area}}}}{{{\text{Wounded}}\;{\text{area}}}} \times 100\% $$

The migration ability of the transfected cells was also evaluated using transwell chambers with 8.0 μm pore sized, gelatinized polycarbonate membrane. The upper transwell chambers were pre-treated with serum-free medium at 37 °C with 5% CO_2_ in incubator for 2 h. The transfected cells were starved with serum-free medium for 12 h, and then seeded in the upper transwell chambers (1.2 × 10^5^ cells per well). At the same time, the lower transwell chambers were added with fresh medium containing 10% FBS, followed by incubating the transwell system for 6 h. The upper chambers were washed twice with 0.01 M PBS (pH = 7.4) and fixed with 4% paraformaldehyde/PBS (pH = 7.4) at room temperature for 10 min. Sterilized cotton swabs were used to remove the cells inside the chambers that didn’t pass through the 8.0 μm pore. The cells on the lower surface of the upper chambers were stained with eosin at 37 °C for 8 min. Migrating cells were observed under an inverted fluorescent microscope, and the number of migrated cells was counted by Image-Pro Plus 6.0 software.

### Capillary-like tube formation

The formation ability of capillary-like tube structure was evaluated by HUVECs in vitro. Matrigel was dissolved at 4 °C overnight, then each well of the pre-cooling 96-well plate was coated with 50 μL growth factor-reduced Matrigel, and followed by incubation at 37 °C for 1 h. Subsequently, HUVECs were transfected with various REDV-TAT-NLS-H_n_/pZNF580 complexes. The transfected cells were trypsinized and seeded on the Matrigel (4 × 10^4^ cells per well), followed by being cultured for 6 h. The cells treated with pZNF580 were used as the negative control. Images of the formation of capillary-like structure at five randomly fields were obtained by using a microscope. The number of the formed tubes in each image was counted manually.

### Western blot analysis

HUVECs were plated on a 6-well plate and transfected with REDV-TAT-NLS-H_n_/pZNF580 complexes for 24 h. The transfected cells were washed three times with cold 0.01 M PBS (pH = 7.4), and followed by extracting the total protein with RIPA lysis buffer containing 1% volume of PMSF. After 30 min on ice, the lysates were centrifuged at 12,000 rpm at 4 °C for 10 min. The total protein was quantified by BCA protein assay kit and denatured by adding 5× SDS. The same amount of each sample (approximately 80 μg protein) was loaded into each lane, separated by 10% polyacrylamide SDS-PAGE gel and transferred onto polyvinylidene difluoride (PVDF) membranes. The membranes were cultured with 8% defat milk in TBST for 1 h and incubated with rabbit anti-ZNF580 polyclonal antibody in TBST overnight. Thereafter, blots were incubated with horseradish peroxidase conjugated anti-rabbit secondary antibody for 1 h and washed twice with TBST. Protein bands were developed by a standard enhanced chemiluminescence (ECL) kit, and observed via a gel image analysis system.

### Quantitative real-time PCR assay

HUVECs were treated with various REDV-TAT-NLS-H_n_/pZNF580 complexes and cultured in an incubator. The cells which were treated with pZNF580 were used as a negative control. After 24 h transfection, the total RNA was extracted from cells with TRIzol reagent, and reverse-transcribed into cDNA using TransScript First-Strand cDNA Synthesis SuperMix. The resulting cDNAs were used as templates for quantitative real-time PCR using TransStart™ Top Green qPCR SuperMix, and detected with a SYBR Green on ABI 7300 stepone sequence detection PCR system (Applied Biosystems). The PCR primer sequences of ZNF580 were as follows: ZNF580 forward 5′-AAAAAGCTTGTGGAGGCGCACGTGCTG-3′, and ZNF580 reverse 5′-AAAAAGATCTTGCCCGGAGTGCGCCCGTG-3′. The expression of glyceraldehyde-3-phosphate dehydrogenase (GAPDH) was used as an internal control. The forward and reverse primer sequences of GAPDH were 5′-AGGTGAAGGTCGGAGTCAAC-3′, 5′-CGCTCCTGGAAGATGGTGAT-3′, respectively. The results were analyzed using StepOne software v2.1.

### Cellular uptake and confocal laser scanning microscopy (CLSM) assay

The cellular uptake and mean fluorescence intensity (MFI) in HUVECs were quantitatively evaluated by a flow cytometry. Cy5-oligonucleotide was mixed with unlabeled oligonucleotide at a 1:1 ratio. Cells were seeded into 6-well plates at 3 × 10^5^ cells per well and transfected with various REDV-TAT-NLS-H_n_/Cy5-oligonucleotide complexes. After 4 h incubation, cells were washed three times with 0.01 M PBS (pH = 7.4) and trypsinized with 0.25% trypsin. Subsequently, the cells were centrifuged, collected and re-suspended in 300 μL PBS (pH = 7.4), followed by analyzing with a flow cytometer (Beckman MoFlo XDP, USA). HUASMCs treated with REDV-TAT-NLS-H_12_/Cy5-oligonucleotide complexes was used as a control group.

CLSM was used to visually observe the intracellular distribution of different REDV-TAT-NLS-H_n_/Cy5-oligonucleotide complexes. For live-cell imaging, cells were seeded in a confocal dish with a density of 1 × 10^5^ cells and cultured for 24 h. Thereafter, cells were transfected by various REDV-TAT-NLS-H_n_/Cy5-oligonucleotide complexes as described above. After 24 h incubation, the cells were washed twice with PBS (pH = 7.4). The lysosomes of HUVECs were stained with the pH sensitive dye LysoTracker Green (DND-26, Life Technologies) at 75 nM for 1 h, and nuclei were stained with Hoechst 33342 for 20 min. Then, the cells were washed twice with PBS (pH = 7.4) and observed by CLSM (Olympus FV1000, Japan) at excitation wavelengths of 649, 504 and 350 nm for Cy5 (red), LysoTracker Green (green) and Hoechst 33342 (blue), respectively. The co-localization rate (CLR) was calculated by Image-Pro Plus 6.0 software according to the following equation.

$$ {\text{Co-localization}}\;{\text{ratio}} = \frac{{{\text{Number}}\;{\text{of}}\;{\text{yellow}}\;{\text{or}}\;{\text{pink}}\;{\text{pixels}}}}{{{\text{Number}}\;{\text{of}}\;{\text{yellow,}}\;{\text{red}}\;{\text{and pink}}\;{\text{pixels}}}} \times 100\% $$

where red corresponds to the Cy5-oligonucleotide in cytoplasm, yellow corresponds to the Cy5-oligonucleotide in endosome/lysosomes, and pink corresponds to the Cy5-oligonucleotide in nucleus.

### Cellular uptake mechanism

To further elucidate the cellular uptake mechanism of REDV-TAT-NLS-H_n_/Cy5-oligonucleotide complexes, HUVECs were pretreated with different inhibitors before transfection [53]. Briefly, cells were seeded onto the 6-well plates and cultured as described above. To probe the cellular uptake mechanism of REDV-TAT-NLS-H_n_/Cy5-oligonucleotide complexes, the cells were pretreated in DMEM with various endocytic inhibitors including chlorpromazine (30 μM), amiloride hydrochloride (30 μM) and filipin III (5 μg mL^−1^) at 37 °C for 1 h, which were used to inhibit the clathrin-mediated endocytosis, micropinocytosis and caveolae-mediated endocytosis, respectively. REDV peptide was also used to pretreat the cells and cultured for 1 h before transfection in order to evaluate the REDV function in endocytic pathway. The REDV-TAT-NLS-H_12_/Cy5-oligonucleotide complexes were added into each well for 4 h incubation. Subsequently, cells were washed three times with 0.01 M PBS (pH = 7.4) followed by trypsinization and centrifugation. Cells were then re-suspended in 300 μL PBS (pH = 7.4) and analyzed by a flow cytometry (Beckman MoFlo XDP, USA).

### In vivo angiogenesis assay

To evaluate the angiogenesis ability of the transfected HUVECs by REDV-TAT-NLS-H_n_/pZNF580 complexes, in vivo angiogenesis assay was performed as previously described [51]. Male mice (6 weeks old, 20–25 g) were used as an animal experimental model. HUVECs were pretreated with different REDV-TAT-NLS-H_n_/pZNF580 complexes for 4 h and cultured for another 24 h. Then the transfected cells were trypsinized with 0.25% trypsin and mixed with 800 μL matrigel at a final concentration of 1 × 10^6^ cells mL^−1^. Before surgery, male mice were treated with chloral hydrate (300 mg/kg) for anesthesia. The mixture was subcutaneously injected in mice using a 1 mL syringe with a 25-gauge needle. Four days later, the mice were injected with excess chloral hydrate to euthanasia. Matrigel implants were removed, fixed with formalin, embedded in paraffin, and sliced into thick sections. Then the sections were stained with hematoxylin and eosin (H&E) and the luminal structures were observed using a microscope. In addition, the sections were immunohistochemically stained with mouse anti-CD31 antibody [diluted in PBS (pH = 7.4) at 1:20] for 60 min and followed with goat anti-mouse IgG H&L secondary antibody (Alexa Fluor^®^ 594). The cell nuclei were stained with DAPI. Immunohistochemically stained sections were used to further determine the formation of microvessel structure which was observed by a fluorescence microscope for each section.

Male mice were preserved by Department of Physiology and Pathophysiology, Logistics University of Chinese People’s Armed Police Force and hosted in SPF room of animal house. All animals were treated following the protocol approved by Armed Police Logistics College and conformed to the “Guide for the protection and use of experimental animals” of the American National Institutes of Health.

### Statistical analysis

All statistical analyses were performed with the one-way ANOVA with a P < 0.05 being considered significant.

## Results

### Particle size and zeta potential of REDV-TAT-NLS-H_n_ micelles and their gene complexes

**Fig. 2:**
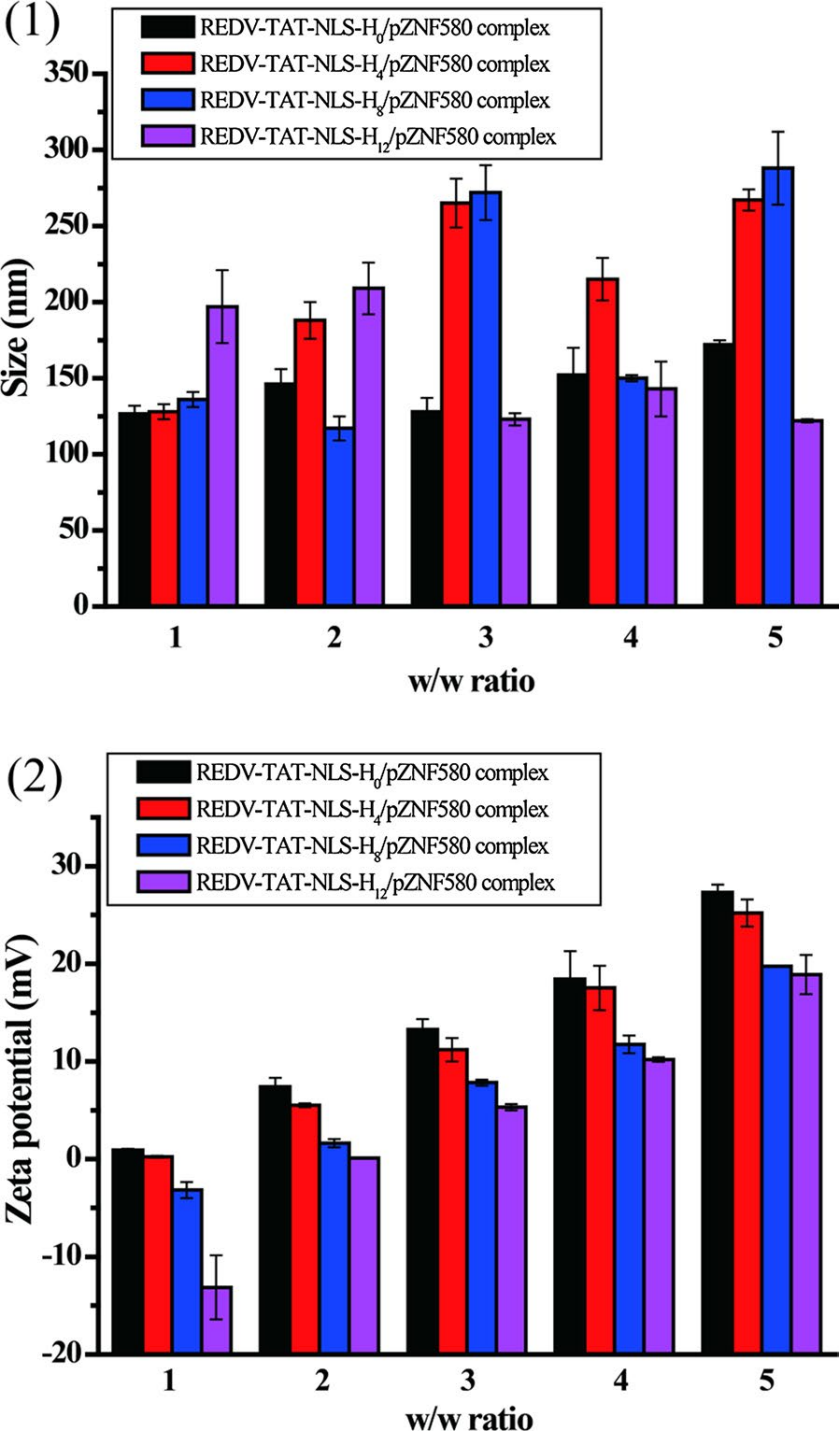
Size and zeta potential of REDV-TAT-NLS-H_n_/pZNF580 complexes at various w/w ratios (1, 2, 3, 4 and 5) of REDV-TAT-NLS-H_n_ and pZNF580. (**1**) Diameter of the REDV-TAT-NLS-H_n_/pZNF580 complexes, (**2**) Zeta potential of the REDV-TAT-NLS-H_n_/pZNF580 complexes

**Fig. 3:**
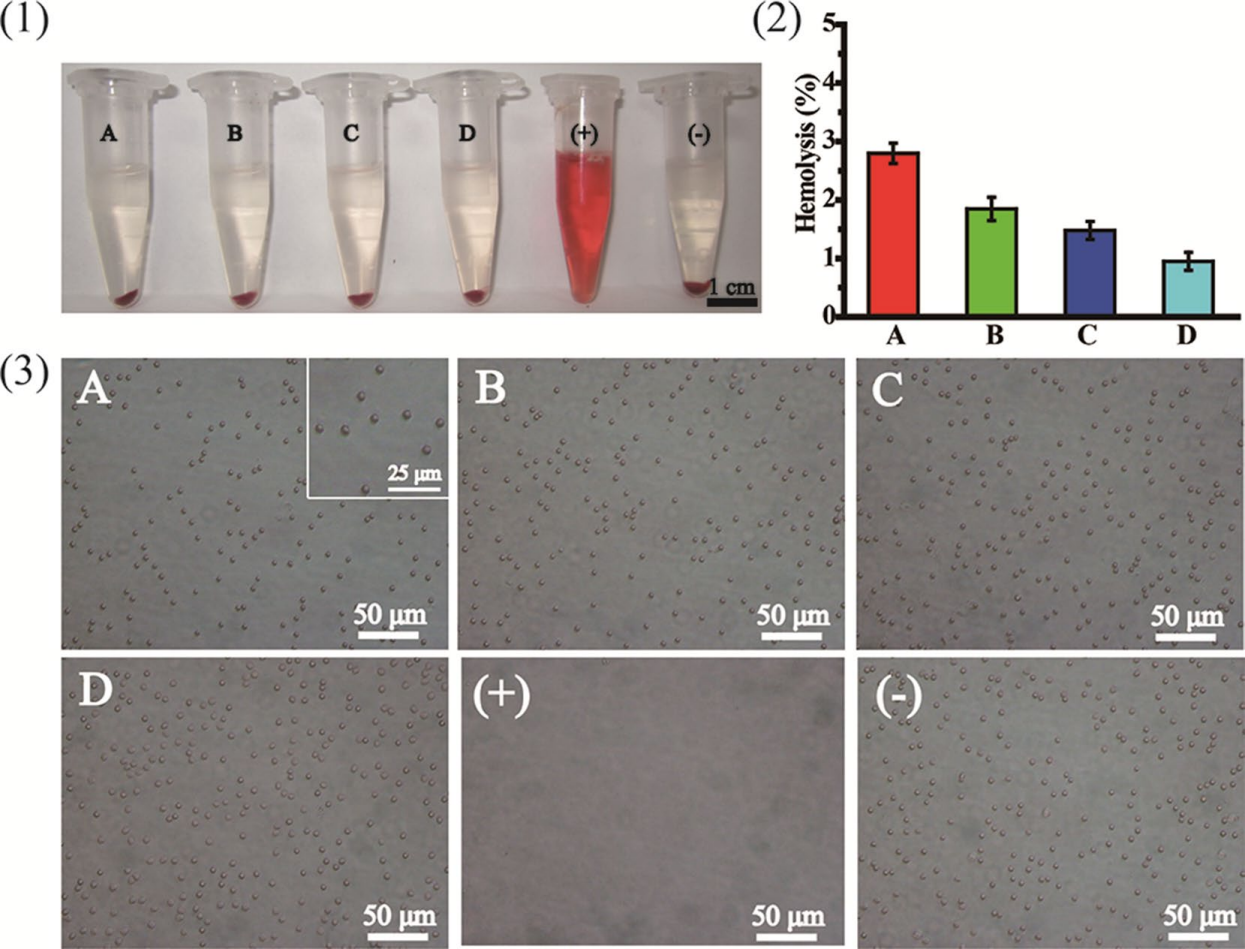
Photographs of the anti-hemolytic activity of REDV-TAT-NLS-H_n_ solution after 24 h incubation (**1**), hemolytic index (**2**), images of RBCs in the presence of REDV-TAT-NLS-H_n_ solution (**3**). (A) REDV-TAT-NLS-H_0_ peptide solution, (B) REDV-TAT-NLS-H_4_ peptide solution, (C) REDV-TAT-NLS-H_8_ peptide solution, (D) REDV-TAT-NLS-H_12_ peptide solution, (+) purified water and (−) PBS

Suitable particle size and surface charge of micelles are beneficial for effective cellular uptake and endocytosis. The size and zeta potential of REDV-TAT-NLS-H_n_ micelles (Table 1) and their pZNF580 complexes with various w/w ratios ranging from 1 to 5 were measured by a Zetasizer Nano ZS (Fig. 3). The REDV-TAT-NLS-H_n_ micelles showed narrower size distribution than their pZNF580 complexes as demonstrated by dynamic light scattering (DLS) curves (Additional file 1: Figure S1–S6). The REDV-TAT-NLS-H_0_ micelles (about 228 nm) were much larger than REDV-TAT-NLS-H_n_ (n = 4, 8, 12) micelles (< 200 nm). Because H residue was hydrophobic at physiological condition, the amphiphilic REDV-TAT-NLS-H_n_ peptides could form stable micelles with relatively compact structure compared with REDV-TAT-NLS-H_0_. The surface charge of these micelles was positive, which was suitable for condensing pZNF580. Moreover, there was no significant distinction in zeta potential of these micelles with different lengths of H_n_ sequence because H_n_ preferentially formed the hydrophobic core of these stable micelles. As shown in Fig. 2(1), the size of REDV-TAT-NLS-H_n_/pZNF580 complexes ranged from 117 to 288 nm, which was suitable for cellular uptake. Furthermore, the zeta potential of the REDV-TAT-NLS-H_n_/pZNF580 complexes increased with increasing w/w ratios from 1 to 5. For REDV-TAT-NLS-H_0_/pZNF580 complexes and REDV-TAT-NLS-H_4_/pZNF580 complexes, their zeta potential was positive when the w/w ratio was 1, while this ratio must be higher than 2 for REDV-TAT-NLS-H_12_/pZNF580 complexes. At the same w/w ratio, the zeta potential of REDV-TAT-NLS-H_n_/pZNF580 complexes decreased with increasing the n number of H_n_ in peptide sequences. This decrease tendency is mainly attributed to the hydrophobic character of H_n_ sequence at physiological condition (pH = 7.4). H_n_ sequence did not induce to further high positive charge [54]. The zeta potential of REDV-TAT-NLS-H_n_/pZNF580 complexes could be mediated by altering the weight ratio of REDV-TAT-NLS-H_n_ peptides and pZNF580, and the sizes of these complexes were less than 300 nm, which were beneficial for endocytosis.

**Table 1:** Size and zeta potential of REDV-TAT-NLS-H_n_ micelles

Sample ID	Size (nm)	*PDI*	Zeta potential (mV)
REDV-TAT-NLS-H_0_	228.1 ± 5.3	0.30 ± 0.04	27.8 ± 1.8
REDV-TAT-NLS-H_4_	142.3 ± 4.3	0.32 ± 0.02	25.8 ± 1.7
REDV-TAT-NLS-H_8_	151.7 ± 2.5	0.30 ± 0.03	26.7 ± 0.4
REDV-TAT-NLS-H_12_	136.6 ± 1.7	0.24 ± 0.06	26.3 ± 0.3

*PDI* polydispersity index

### Hemocompatibility of REDV-TAT-NLS-H_n_ peptides

The hemocompatibility of gene carriers is very important for their in vivo application. To evaluate the hemocompatibility of REDV-TAT-NLS-H_n_ peptides, hemolytic activity and RBC aggregation assay were performed in vitro [55]. RBCs treated with PBS (pH = 7.4) and purified water were used as the negative and positive controls, respectively. As shown in Fig. 3(1), (2), the hemolytic index of REDV-TAT-NLS-H_n_ peptides decreased with increasing H_n_ sequence length. All peptide micelles showed lower hemolysis than standard criteria (5%). According to ISO 10993-4, they can be considered as feasible blood-contacting biomaterials. In addition, the RBC aggregation results also showed that more RBCs remained unchanged when the n number of H_n_ sequences increased from 4 to 12 (Fig. 3(3)). These REDV-TAT-NLS-H_n_ peptides exhibited good hemocompatibility.

### pDNA binding ability of REDV-TAT-NLS-H_n_ peptides

**Fig. 4:**
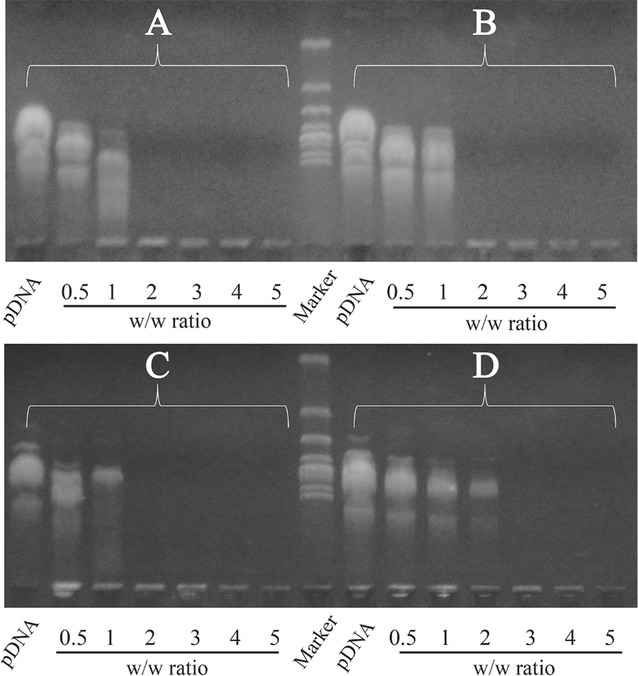
Agarose gel electrophoresis of various REDV-TAT-NLS-H_n_/pZNF580 complexes at different w/w ratios varying from 0.5 to 5: (A) REDV-TAT-NLS-H_0_/pZNF580 complexes, (B) REDV-TAT-NLS-H_4_/pZNF580 complexes, (C) REDV-TAT-NLS-H_8_/pZNF580 complexes, (D) REDV-TAT-NLS-H_12_/pZNF580 complexes

It is well known that DNA binding ability induced by electrostatic interaction is a prerequisite for gene carriers. REDV-TAT-NLS-H_0_, REDV-TAT-NLS-H_4_, REDV-TAT-NLS-H_8_ and REDV-TAT-NLS-H_12_ peptides are positively charged in physiological condition. In this study, the binding and condensing ability of REDV-TAT-NLS-H_n_/pZNF580 complexes with the w/w ratios ranging from 0.5 to 5 was evaluated by agarose gel electrophoresis. The REDV-TAT-NLS-H_n_/pZNF580 complexes were prepared by mixing peptides with pZNF580, and incubated for 30 min. As shown in Fig. 4, the REDV-TAT-NLS-H_0_, REDV-TAT-NLS-H_4_, REDV-TAT-NLS-H_8_ and REDV-TAT-NLS-H_12_ peptides were able to completely retard the mobility of pZNF580 at the w/w ratios of 2, 2, 2 and 3, respectively. Obviously, the condensing pZNF580 ability of these peptides decreased when H_n_ sequence increased from 4 to 12. This trend was consistent with the zeta potential results. In a word, all of these peptides showed strong pZNF580 condensing ability. It’s well known that highly positive zeta potential is necessary for gene carriers to condense and deliver DNA efficiently. Therefore, the w/w ratio of 5 was chosen for the following studies.

### In vitro cytotoxicity

**Fig. 5:**
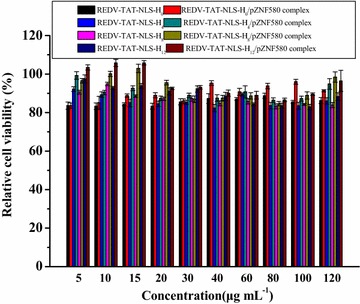
Relative cell viability of HUVECs after 48 h in the presence of different REDV-TAT-NLS-H_n_ or REDV-TAT-NLS-H_n_/pZNF580 complexes with peptide concentration ranging from 5 to 120 μg mL^−1^ at w/w ratio of 5 (mean ± SD, n = 3)

The cytotoxicity of gene carriers is a critical factor for their future application in vivo. The in vitro relative cell viability of REDV-TAT-NLS-H_n_ and REDV-TAT-NLS-H_n_/pZNF580 complexes was evaluated by MTT assay. The concentration of peptides ranged from 5 to 120 μg mL^−1^. As shown in Fig. 5, no significant cytotoxicity was observed in all peptide groups. With the increase of concentration, the relative cell viability did not decrease obviously. At the same concentration, compared with REDV-TAT-NLS-H_n_ (n = 4, 8 and 12) micelles, the REDV-TAT-NLS-H_n_/pZNF580 complexes showed relatively high cell viability. Moreover, all groups exhibited very low cytotoxicity and their relative cell viabilities were higher than 80% at the concentration of 120 mg mL^−1^. These results indicated that these REDV-TAT-NLS-H_n_ (n = 4, 8, 12) peptides would be safe as a gene carrier.

### In vitro transfection

**Fig. 6:**
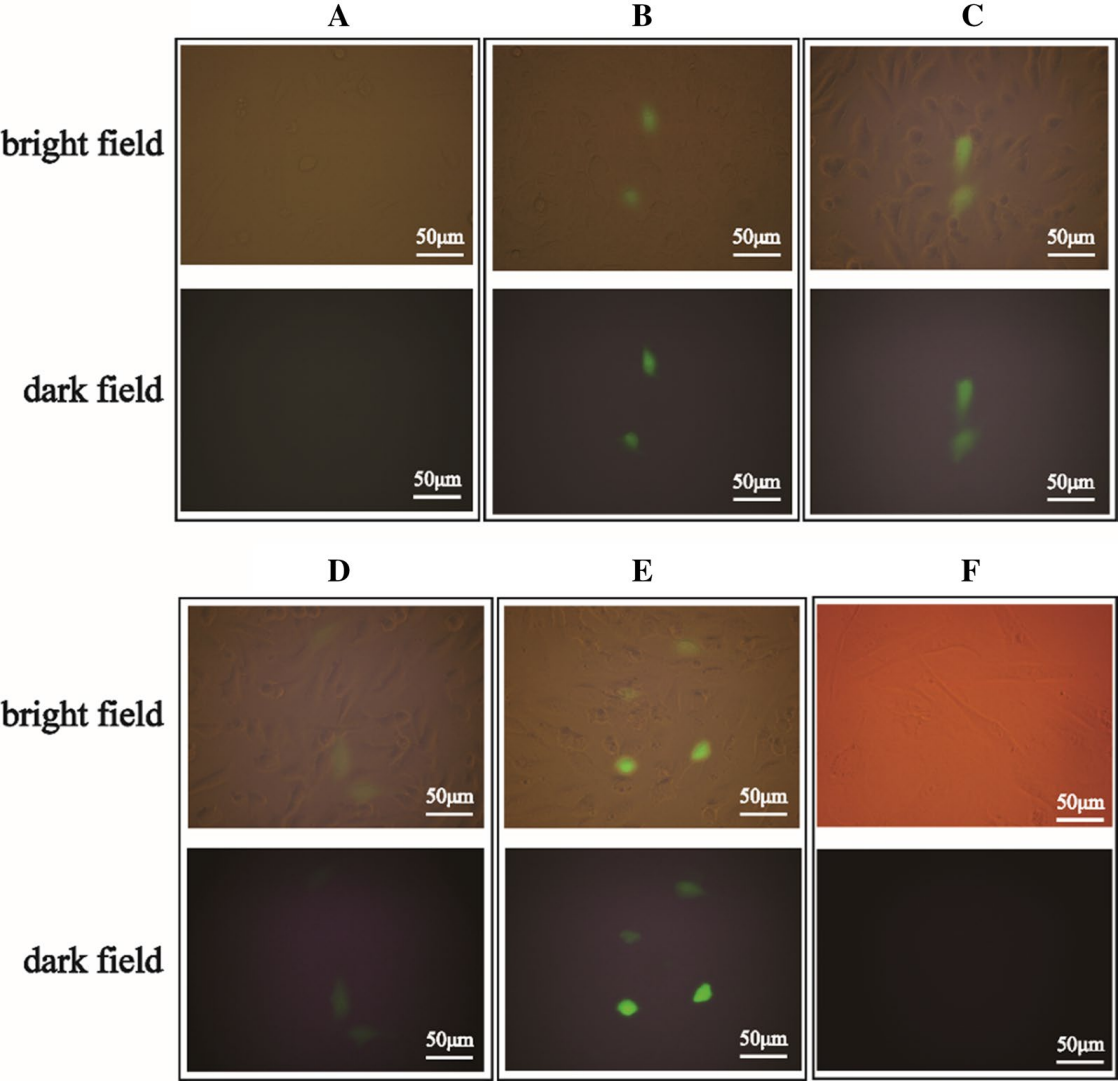
Fluorescence images of HUVECs and HUASMCs transfected by REDV-TAT-NLS-H_n_/pZNF580 complexes at the w/w ratio of 5 with the peptide concentration of 40 μg mL^−1^. **A** HUVECs treated with pZNF580 as a negative control group. **B** HUVECs treated with REDV-TAT-NLS-H_0_/pZNF580 complexes. **C** HUVECs treated with REDV-TAT-NLS-H_4_/pZNF580 complexes. **D** HUVECs treated with REDV-TAT-NLS-H_8_/pZNF580 complexes. **E** HUVECs treated with REDV-TAT-NLS-H_12_/pZNF580 complexes. **F** HUASMCs treated with REDV-TAT-NLS-H_12_/pZNF580 complexes

In order to investigate the influence of introducing H_n_ sequence into gene carriers on gene delivery, HUVECs were transfected by different REDV-TAT-NLS-H_n_/pZNF580 complexes in vitro. The HUVECs transfected with REDV-TAT-NLS-H_0_/pZNF580 complexes and naked pZNF580 gene were used as positive and negative controls, respectively. As illustrated in Fig. 6, all REDV-TAT-NLS-H_n_/pZNF580 complexes groups showed green fluorescence protein, implying efficient gene delivery and successful pZNF580 expression. Among all groups, REDV-TAT-NLS-H_12_/pZNF580 complexes group showed the best transfection result. All REDV-TAT-NLS-H_n_/pZNF580 complexes could deliver pZNF580 into cells to express ZNF580 gene. Compared with the PEI-based gene carriers in our previous studies, the transfection efficiency of REDV-TAT-NLS-H_n_/pZNF580 complexes was low, but their cytotoxicity was very low even at high concentration. Because ECs are hard to be transfected by gene complexes [56]. The instability and limited positive charges of the peptide-based gene carriers induced low transfection efficiency. In addition, HUASMCs were treated with REDV-TAT-NLS-H_12_/pZNF580 complexes and showed very low transfection, which proved the target function of REDV-TAT-NLS-H_12_/pZNF580 complexes for HUVECs.

### Cell migration assay

**Fig. 7:**
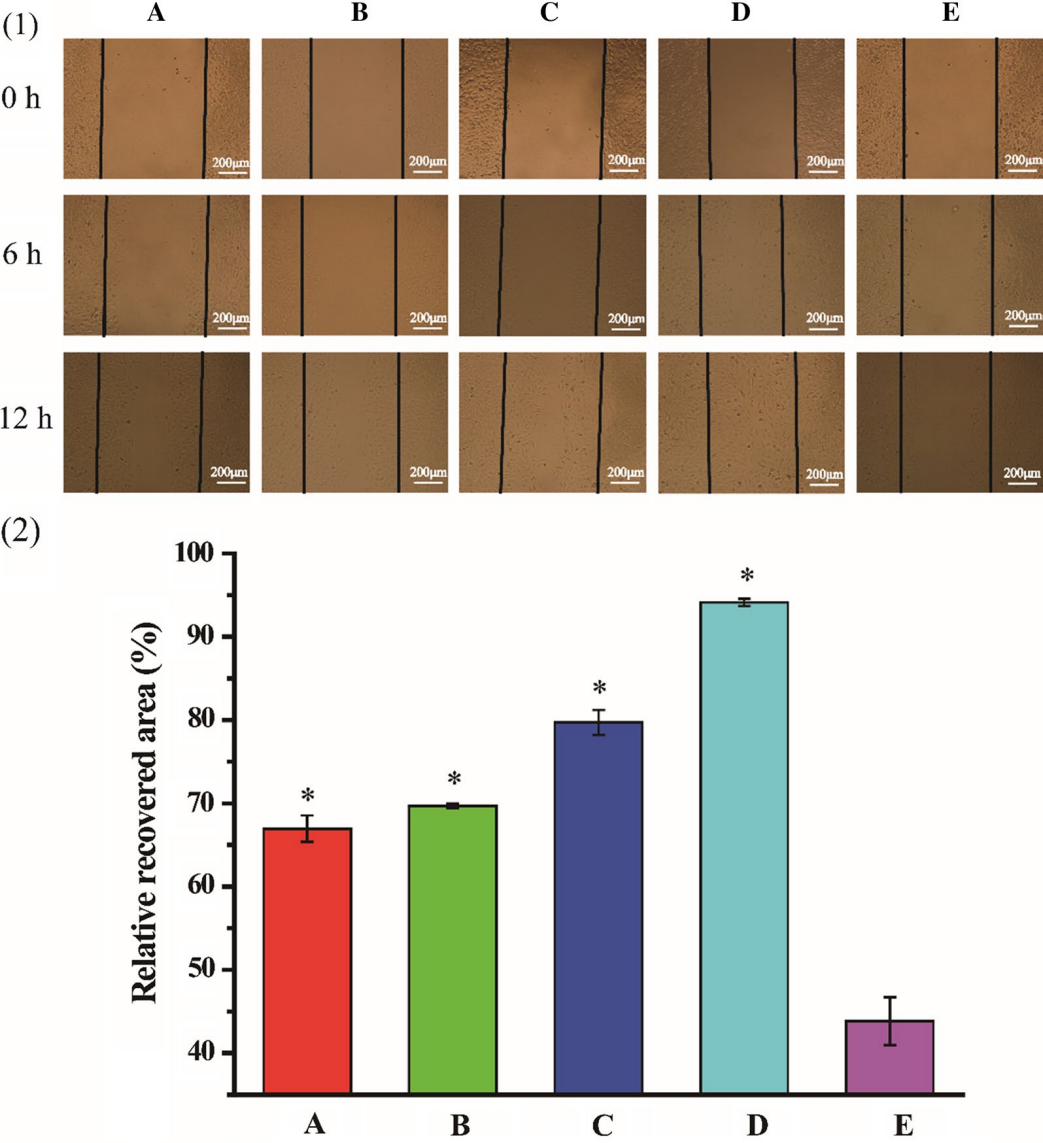
Recovered process of HUVECs at different time points (**1**) and the relative recovered area after 12 h calculated by Image-J software (**2**). (A) HUVECs treated with REDV-TAT-NLS-H_0_/pZNF580 complexes, (B) HUVECs treated with REDV-TAT-NLS-H_4_/pZNF580 complexes, (C) HUVECs treated with REDV-TAT-NLS-H_8_/pZNF580 complexes, (D) HUVECs treated with REDV-TAT-NLS-H_12_/pZNF580 complexes, (E) HUVECs treated with pZNF580 as a control group (mean ± SD, n = 3, *P < 0.05 vs. control group)

The migration ability of HUVECs plays an important role in the formation of a confluent EC monolayer for cardiovascular disease treatment. Here, wound healing assay was used to evaluate the migration ability of transfected HUVECs by REDV-TAT-NLS-H_n_/pZNF580 complexes. After 24 h incubation, an artificial scratch with parallel borders was mechanically created and migration process at different time points was monitored by capturing pictures (Fig. 7(1)). HUVECs treated with REDV-TAT-NLS-H_0_/pZNF580 complexes and naked pZNF580 gene were used as positive and negative controls, respectively. The migration rate of transfected HUVECs with REDV-TAT-NLS-H_n_/pZNF580 complexes was much higher than the negative control group (Fig. 7). REDV-TAT-NLS-H_12_/pZNF580 complexes group showed the highest migration rate (94.13 ± 0.43%). The complexes containing H_n_ sequence were beneficial for cell transfection and migration.

**Fig. 8:**
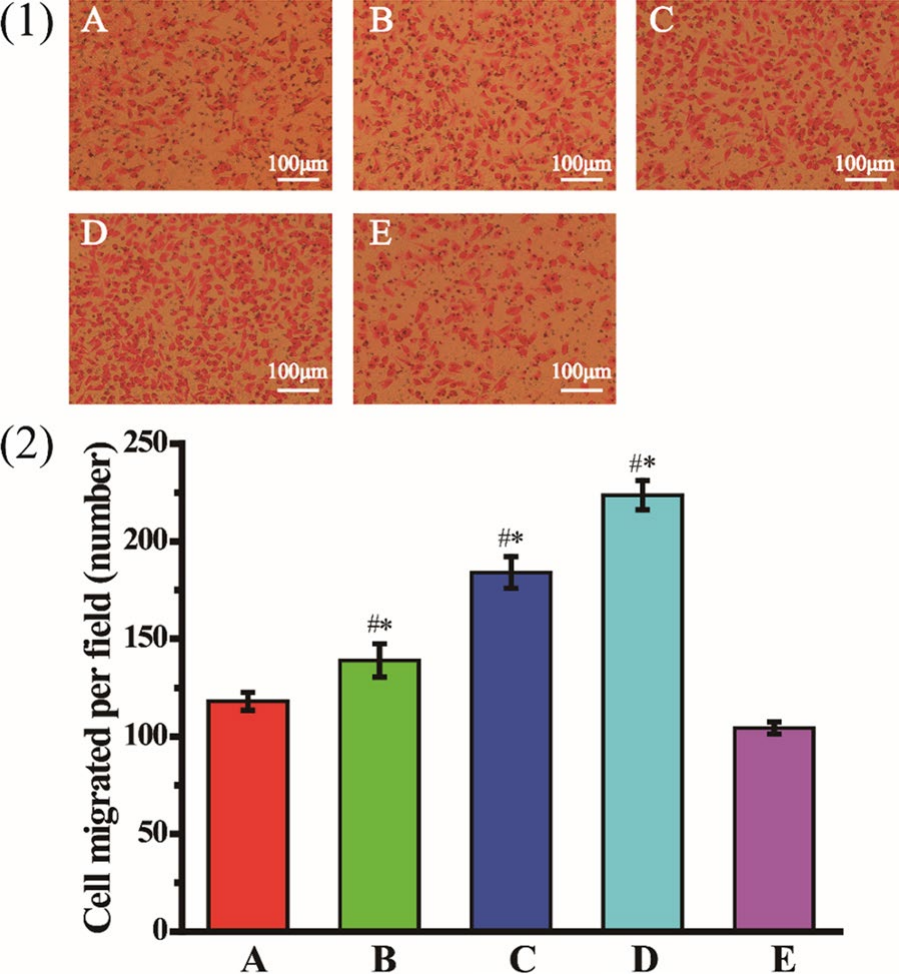
Migration assay of HUVECs through a transwell chamber over 6 h (**1**) and the average number of cells migrating (**2**). (A) HUVECs treated with REDV-TAT-NLS-H_0_/pZNF580 complexes, (B) HUVECs treated with REDV-TAT-NLS-H_4_/pZNF580 complexes, (C) HUVECs treated with REDV-TAT-NLS-H_8_/pZNF580 complexes, (D) HUVECs treated with REDV-TAT-NLS-H_12_/pZNF580 complexes, (E) ECs treated with pZNF580 as a control group (mean ± SD, n = 3, *P < 0.05 vs. control group). ^#^*P* < 0.05 vs. group A

In addition, the cell migration ability was also evaluated by transwell chambers. As shown in Fig. 8, REDV-TAT-NLS-H_n_/pZNF580 complexes groups showed larger cell migration number than the control group. The cell migration number of REDV-TAT-NLS-H_12_/pZNF580 complexes group was the largest among all groups (Fig. 8(D)). The results of transwell migration assay is approximately similar to wound healing assay. REDV-TAT-NLS-H_n_/pZNF580 complexes containing long H_n_ sequence benefited for high cell migration and proliferation. H_n_ sequence can be protonated and help to promote the endosome/lysosome escape via proton sponge effect, which further enhances the transfection efficiency.

### In vitro tube formation

**Fig. 9:**
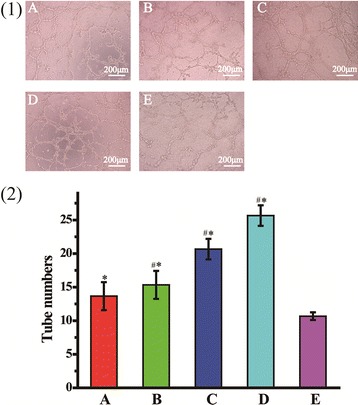
Images of different REDV-TAT-NLS-H_n_/pZNF580 complexes induced in vitro tube formation of HUVECs (**1**) and tube number formed in different group (**2**). (A) REDV-TAT-NLS-H_0_/pZNF580 complexes treated group, (B) REDV-TAT-NLS-H_4_/pZNF580 complexes treated group, (C) REDV-TAT-NLS-H_8_/pZNF580 complexes treated group, (D) REDV-TAT-NLS-H_12_/pZNF580 complexes treated group, (E) pZNF580 treated group as a control group (mean ± SD, n = 3, *P < 0.05 vs. control group). ^#^*P* < 0.05 vs. group A

The formation of capillary-like tubes via HUVECs migration toward each other is an integral part of numerous pathologies [57]. Here, angiogenesis assay was used to evaluate the tube formation ability of transfected HUVECs. Without any growth factors, the transfected HUVECs proliferated and migrated on matrigel to form vascular rings (Fig. 9). HUVECs treated with REDV-TAT-NLS-H_0_/pZNF580 complexes and naked pZNF580 were used as the positive and negative controls, respectively. After 6 h incubation, tube formation can be observed in all groups. Compared with the negative group, HUVECs treated with REDV-TAT-NLS-H_n_/pZNF580 complexes showed a strong angiogenesis ability, especially the REDV-TAT-NLS-H_12_/pZNF580 complexes group with a tube number of nearly 26 (Fig. 9(D)). This group exhibited approximately twofold tubes compared with the positive control (Fig. 9(A)), while REDV-TAT-NLS-H_4_/pZNF580 complexes and REDV-TAT-NLS-H_8_/pZNF580 complexes groups formed relatively less tubes. These results indicated that REDV-TAT-NLS-H_12_/pZNF580 complexes significantly enhanced tube-forming ability. It is owing to longer H_n_ sequence feasible to promote gene delivery efficiency. The expression of pZNF580 plasmid in nucleus enhanced the proliferation, migration as well as vascularization of HUVECs.

### Western blot analysis

**Fig. 10:**
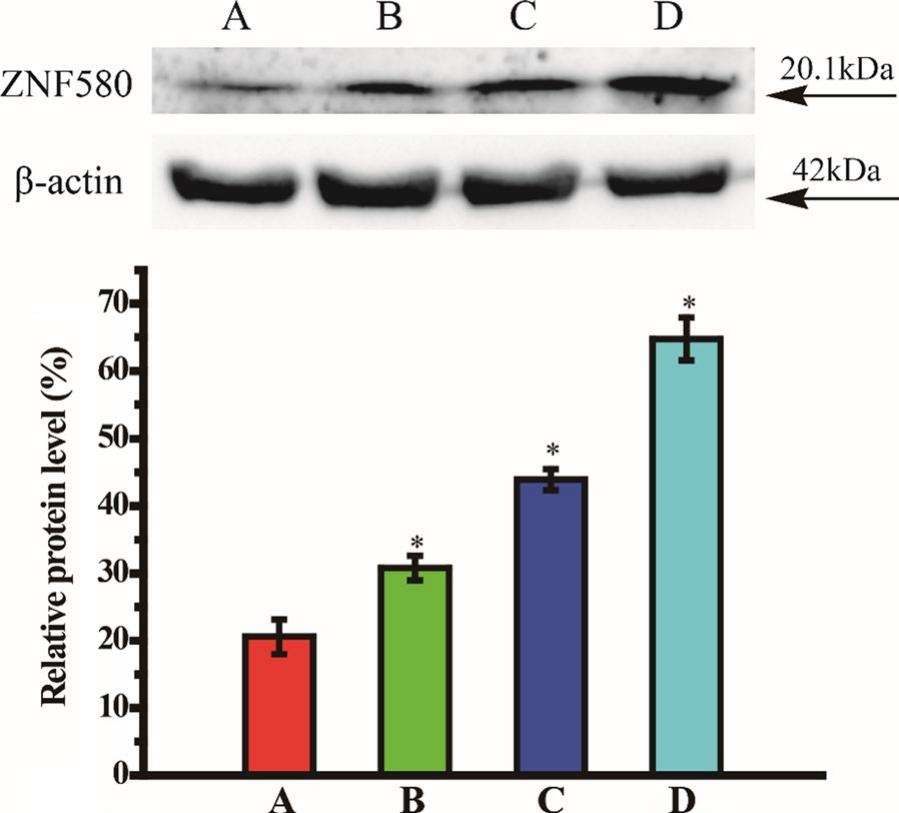
The western blot analysis of ZNF580 protein expression in HUVECs treated with different REDV-TAT-NLS-H_n_/pZNF580 complexes. (A) REDV-TAT-NLS-H_0_/pZNF580 complexes treated group, (B) REDV-TAT-NLS-H_4_/pZNF580 complexes treated group, (C) REDV-TAT-NLS-H_8_/pZNF580 complexes treated group, (D) REDV-TAT-NLS-H_12_/pZNF580 complexes treated group (mean ± SD, n = 3, *P < 0.05 vs. control group A)

Western blot analysis is a powerful method to quantitatively analyze the expression of ZNF580 gene in HUVECs. HUVECs was treated with REDV-TAT-NLS-H_0_/pZNF580 complexes, which was used as a positive group. Relative protein level (%) was calculated by comparing the protein expression of ZNF580 gene with β-actin gene. As shown in Fig. 10, compared with REDV-TAT-NLS-H_0_/pZNF580 complexes group, the cells which were treated with REDV-TAT-NLS-H_n_/pZNF580 complexes exhibited higher relative protein level. Cells transfected with REDV-TAT-NLS-H_12_/pZNF580 complexes showed a relative protein level of 64.74%, which was the highest protein expression among all groups. After endosomal/lysosomal escape through the buffer capability of H_n_ sequence, NLS sequence in REDV-TAT-NLS-H_12_/pZNF580 complexes enhanced the nuclear location ability of pZNF580. Therefore, the peptide-based gene carriers, especially REDV-TAT-NLS-H_12_, could efficiently transfect HUVECs, improved the escape ability from endosome/lysosome, and enhanced the gene expression level successfully.

### Quantitative real-time RT-PCR assay

**Fig. 11:**
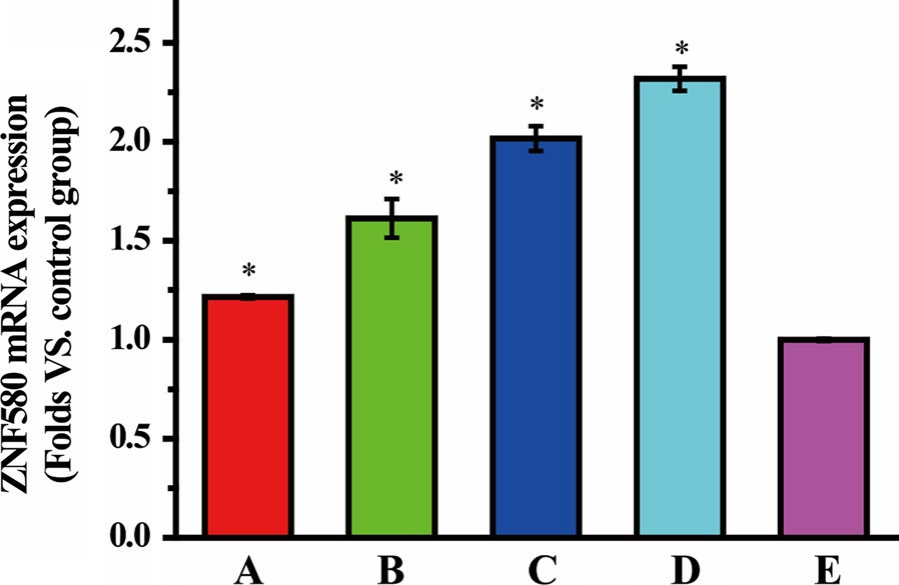
Quantitative mRNA expression of HUVECs transfected by different REDV-TAT-NLS-H_n_/pZNF580 complexes. (A) REDV-TAT-NLS-H_0_/pZNF580 complexes treated group, (B) REDV-TAT-NLS-H_4_/pZNF580 complexes treated group, (C) REDV-TAT-NLS-H_8_/pZNF580 complexes treated group, (D) REDV-TAT-NLS-H_12_/pZNF580 complexes treated group, (E) pZNF580 treated group as a control group (mean ± SD, n = 3, *P < 0.05 vs. control group)

The expression of ZNF580 mRNA in transfected HUVECs was measured by Real-Time RT-PCR analysis technique. The results of ZNF580 mRNA expression were shown in Fig. 11. The relative ZNF580 mRNA expression levels of REDV-TAT-NLS-H_0_/pZNF580, REDV-TAT-NLS-H_4_/pZNF580, REDV-TAT-NLS-H_8_/pZNF580 and REDV-TAT-NLS-H_12_/pZNF580 complexes groups were much higher than the control group (Fig. 11(E)). Compared with REDV-TAT-NLS-H_0_/pZNF580 complexes group, the HUVECs treated with REDV-TAT-NLS-H_n_/pZNF580 complexes showed high ZNF580 mRNA expression level. REDV-TAT-NLS-H_12_/pZNF580 complexes group expressed highest ZNF580 mRNA, which highlights the effect of H_n_ sequence in gene carriers. These results proved that the efficient gene delivery of REDV-TAT-NLS-H_n_/pZNF580 complexes in mRNA level was similar to the results of western blot analysis.

### Cellular uptake and CLSM

**Fig. 12:**
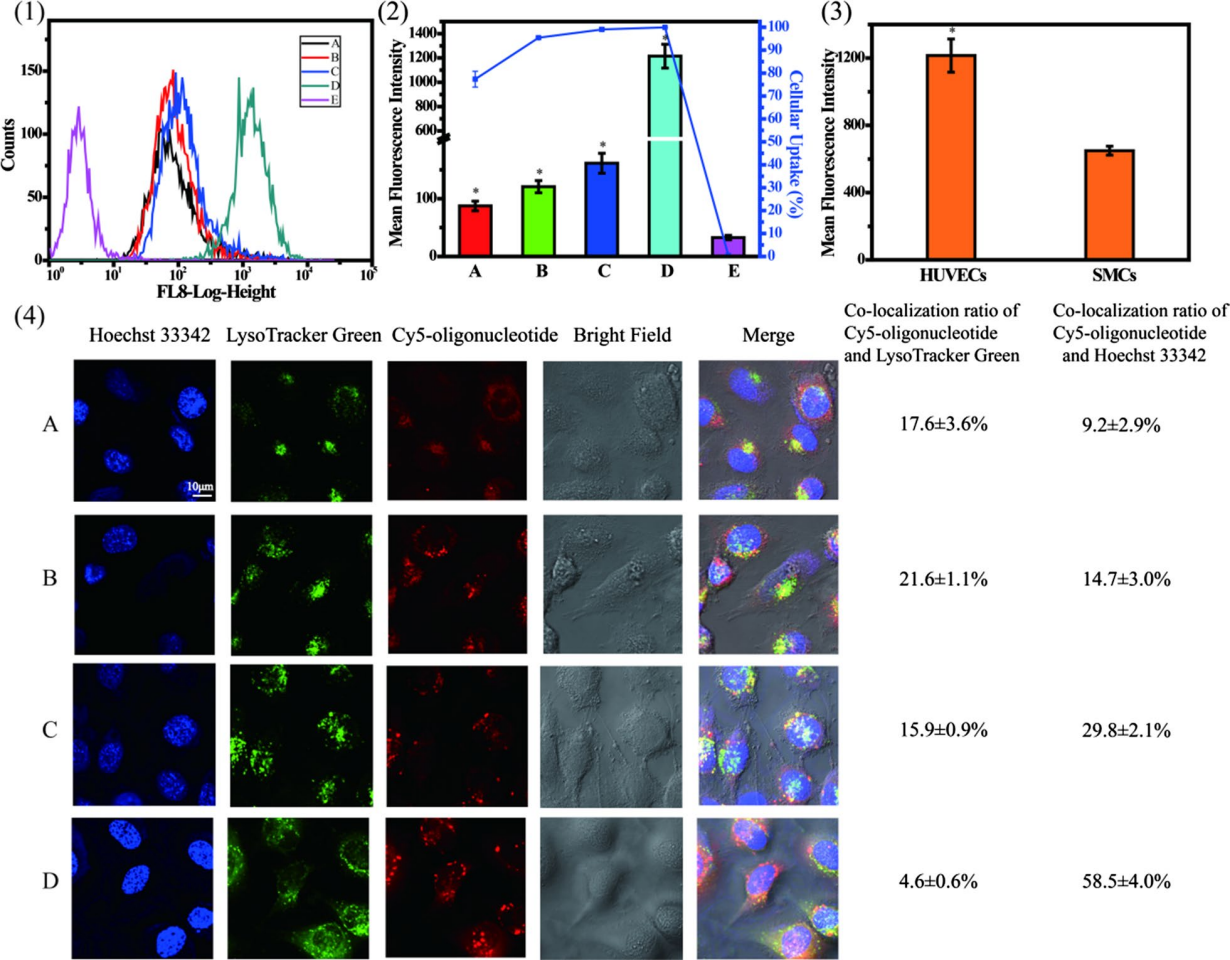
Cellular uptake analysis of various REDV-TAT-NLS-H_n_/Cy5-oligonucleotide complexes after 4 h transfection (**1**), percentages of cellular uptake and mean fluorescence intensity (MFI) measured by flow cytometry (**2**), mean fluorescence intensity of HUVECs and HUASMCs transfected by REDV-TAT-NLS-H_12_/Cy5-oligonucleotide complexes (**3**), and CLSM images of intracellular trafficking of various REDV-TAT-NLS-H_n_/Cy5-oligonucleotide complexes (**4**). (A) REDV-TAT-NLS-H_0_/Cy5-oligonucleotide complexes treated group, (B) REDV-TAT-NLS-H_4_/Cy5-oligonucleotide complexes treated group, (C) REDV-TAT-NLS-H_8_/Cy5-oligonucleotide complexes treated group, (D) REDV-TAT-NLS-H_12_/Cy5-oligonucleotide complexes treated group, (E) Cy5-oligonucleotide treated group as a control group (mean ± SD, n = 3, *P < 0.05 vs. control group)

To evaluate the cellular uptake capability of these complexes in HUVECs, different gene complexes were cultured with cells for 4 h and detected by a flow cytometry. As shown in Fig. 12, the cellular uptake of gene complexes with H_n_ sequence was above 95% and much higher than that of REDV-TAT-NLS-H_0_/Cy5-oligonucleotide complexes. The MFI of REDV-TAT-NLS-H_n_/Cy5-oligonucleotide complexes groups also showed a similar tendency. The MFI values increased along with H_n_ sequence length increased from 4 to 12. Especially, the REDV-TAT-NLS-H_12_/Cy5-oligonucleotide complexes group demonstrated significantly high MFI value (MFI = 1215), which was nearly 14 folds to REDV-TAT-NLS-H_0_/Cy5-oligonucleotide complexes group. This phenomenon could be interpreted that the H_n_ sequence was hydrophobic under neutral pH condition, which facilitated hydrophobic interaction of gene carrier with cell membrane so as to promote cellular uptake [58].

Besides, the MFI of transfected HUASMCs was also evaluated to verify the target function of the gene complexes. In Fig. 12(3), the results showed that the MFI of transfected HUVECs was significantly higher than in HUASMCs, which demonstrated the targeting ability of REDV-TAT-NLS-H_12_/Cy5-oligonucleotide complexes to HUVECs.

Furthermore, the intercellular distribution of Cy5-oligonucleotide in transfected HUVECs was evaluated by CLSM. The intracellular yellow pixels represented the entrapment of REDV-TAT-NLS-H_n_/Cy5-oligonucleotide complexes in the endosome/lysosome, whereas the intracellular pink pixels illustrated their presence in nucleus. As shown in Fig. 12(4), the nucleus CLR of REDV-TAT-NLS-H_n_/Cy5-oligonucleotide complexes increased with increasing H_n_ sequence length, while the lysosome CLR decreased. These results demonstrated that more complexes with longer H_n_ sequence entered into HUVECs, escaped from endosome/lysosomes into cytoplasm and entered into nucleus. As aforementioned, the H_n_ sequence was hydrophobic at pH 7.4, which was advantageous for cellular uptake. Besides, this H_n_ sequence could change to hydrophilic and positive-charged under acid condition in endosome/lysosome. Thus, it could help endosomal/lysosomal membrane rupture and escape owing to the pH buffer capacity of imidazole ring in H_n_ residues [59, 60]. In addition, the nucleus CLR of REDV-TAT-NLS-H_12_/Cy5-oligonucleotide complexes was 58.5 ± 4.0%, which was about sixfold as high as REDV-TAT-NLS-H_0_/Cy5-oligonucleotide complexes group. These results indicated that these peptides containing H_n_, REDV and NLS sequences, especially the REDV-TAT-NLS-H_12_ peptide can promote the cellular uptake efficiency, endosome/lysosome escape ability and nuclear location capacity, which could improve the efficiency of gene delivery.

### Cellular uptake mechanism

Gene carriers can enter into cells via several pathways, such as micropinocytosis, clathrin-mediated endocytosis, caveolae-mediated endocytosis and other endocytic pathways [61–64]. To study the cellular uptake mechanism of these REDV-TAT-NLS-H_n_/pZNF580 complexes, different inhibitors were used for particular endocytic pathways. In this paper, three typical endocytosis pathways including clathrin-mediated endocytosis, caveolae-mediated endocytosis and micropinocytosis were studied. Amiloride (Amil) is reported to inhibit the micropinocytosis by blocking the Na^+^/H^+^ channels, chlorpromazine (CPZ) can inhibit clathrin-mediated endocytosis by the interruption of clathrin, while filipin (Filip) can inhibit the caveolae-mediated endocytosis via combination with cholesterol [65].

**Fig. 13:**
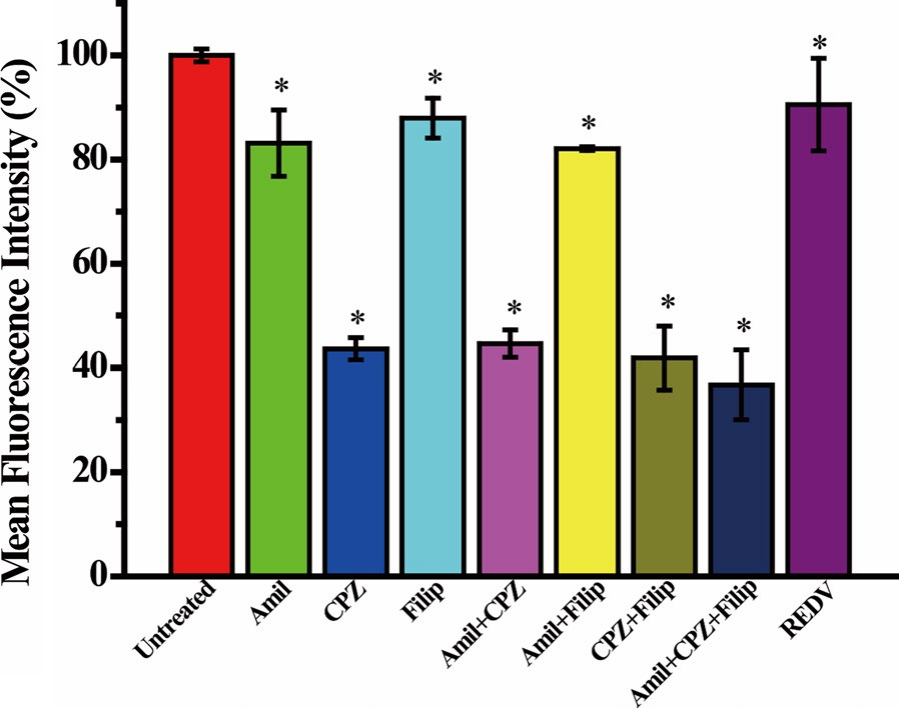
Effects of endocytic inhibitors on cellular uptake of REDV-TAT-NLS-H_12_/Cy5-oligonucleotide complexes on HUVECs. HUVECs treated with REDV-TAT-NLS-H_12_/Cy5-oligonucleotide complexes without inhibitors as a control group (mean ± SD, n = 3, *P < 0.05 vs. control group)

Herein, because of the best results of cellular uptake and intercellular distribution, REDV-TAT-NLS-H_12_/Cy5-oligonucleotide complexes were used to transfect HUVECs to investigate the cellular uptake mechanism. As shown in Fig. 13, a great reduction (about 56%) occurred when HUVECs were cultured with CPZ for 1 h before transfection. The cellular uptake reduced approximately 17 and 12% when HUVECs were pretreated with Amil and Filip, respectively. These results demonstrated that clathrin-mediated endocytosis is the main pathway for cellular uptake of REDV-TAT-NLS-H_12_/Cy5-oligonucleotide complexes. In addition, the caveolae-mediated endocytosis and micropinocytosis were also responsible for the cellular uptake of REDV-TAT-NLS-H_12_/Cy5-oligonucleotide complexes, but they were not the main pathways. The addition of two or three inhibitors in one test group was performed to evaluate the effect of the other pathways. When cells were pre-incubated with these three inhibitors before transfection, the cellular uptake of REDV-TAT-NLS-H_12_/Cy5-oligonucleotide complexes still remained 36.8%. In addition, the cellular uptake of only REDV treated group decreased almost 10%, which means that the targeting REDV peptide contributed to the cellular uptake (10%) of REDV-TAT-NLS-H_12_/Cy5-oligonucleotide complexes. The above results indicated that some other endocytic pathways existed because the cellular uptake still remained about 27% except for three inhibitors and REDV mediated endocytosis. In conclusion, the cellular uptake mechanism was multiple action of different endocytosis pathways, and the clathrin-mediated endocytosis was the major internalization pathway of REDV-TAT-NLS-H_12_/Cy5-oligonucleotide complexes in HUVECs.

### In vivo angiogenesis assay

**Fig. 14:**
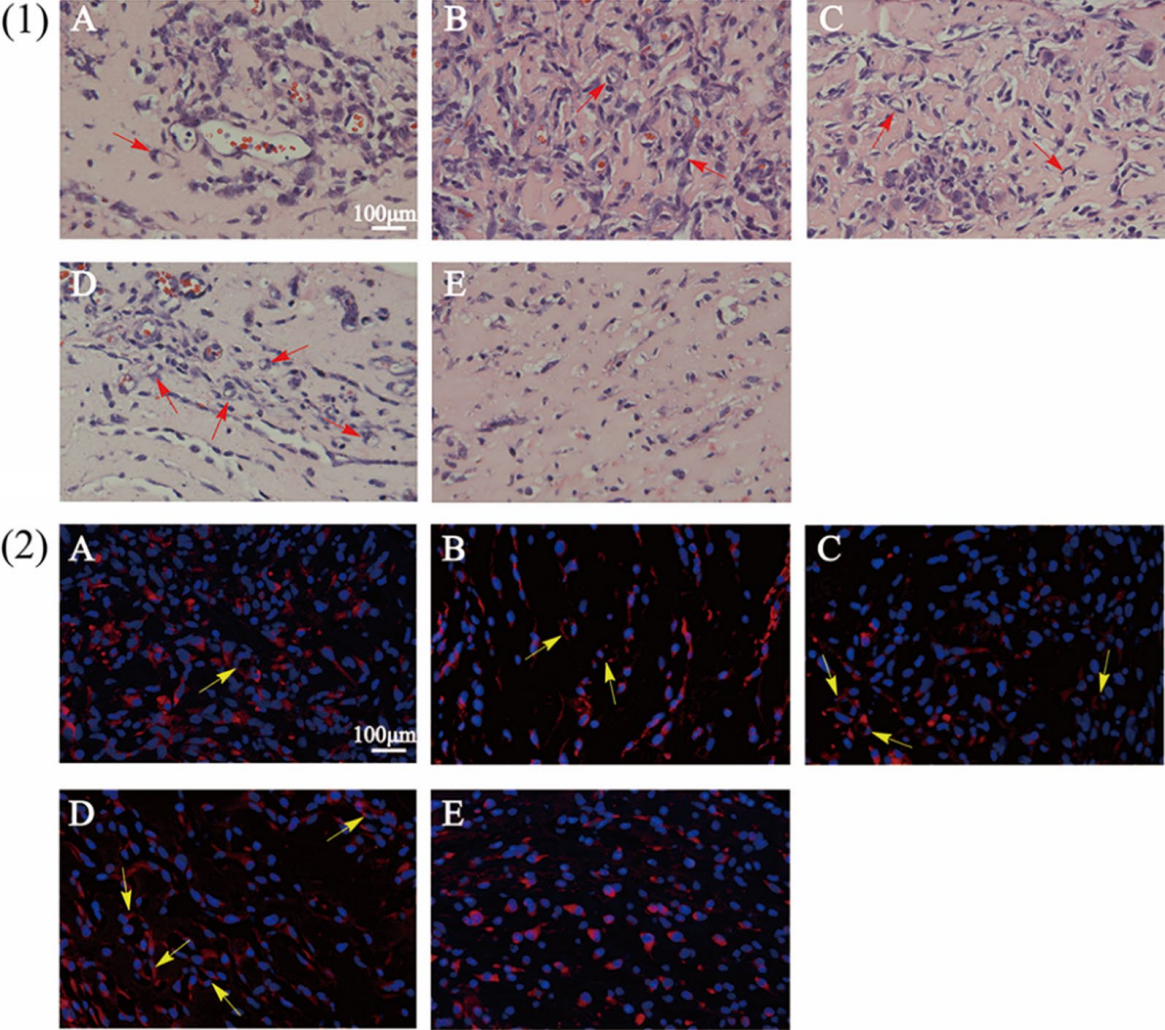
Neovascularization assay evaluating in vivo tube formation of HUVECs treated with REDV-TAT-NLS-H_n_/pZNF580 complexes. (A) REDV-TAT-NLS-H_0_/pZNF580 complexes treated group, (B) REDV-TAT-NLS-H_4_/pZNF580 complexes treated group, (C) REDV-TAT-NLS-H_8_/pZNF580 complexes treated group, (D) REDV-TAT-NLS-H_12_/pZNF580 complexes treated group, (E) pZNF580 treated group. Implants were sectioned and stained with hematoxylin and eosin (H&E) (**1**) and immunohistochemically stained with anti-CD31 antibody (**2**)

To further investigate the angiogenesis ability in vivo, the transfected HUVECs were mixed with Matrigel and subcutaneously implanted into mice. After 4 days, Matrigel implants were removed and sectioned. H&E staining and anti-CD31 staining were performed to reveal the formation of microvessel structure, and the results were shown in Fig. 14. HUVECs, which were transfected with REDV-TAT-NLS-H_n_/pZNF580 complexes, showed obvious microvessel structures. A great number of luminal structures were observed in the implants containing REDV-TAT-NLS-H_12_/pZNF580 complexes transfected cells compared with REDV-TAT-NLS-H_0_/pZNF580 complexes group (Fig. 14(A), (D)). In contrast, the cells only treated with pZNF580 showed few microvessel structures (Fig. 14(E)), which means its poor tube formation ability. To further characterize the microvessel structure clearly, Fig. 14(2) showed the images of immunofluorescence staining with anti-CD31 for HUVECs. These results indicated that the expression pZNF580 could promote angiogenesis in vivo. In consistent with the results of the in vitro tube formation assay, the REDV-TAT-NLS-H_12_/pZNF580 complexes could obviously enhance the angiogenesis ability both in vitro and in vivo.

## Discussion

In recent years, gene therapy offers a promising alternative for the treatment of cardiovascular diseases. The development of non-virus gene carriers with low cytotoxicity and high safety is especially necessary for vascularization. Cationic peptides are synthesized from natural amino acids, which are widely used as gene carriers. Compared with other gene carriers, the peptide-based gene carriers exhibit relatively low cytotoxicity and good biocompatibility. As a kind of widely used CPPs, the arginine-rich TAT peptide shows high ability of crossing cell membrane to overcome the first barrier of gene delivery. Herein, three peptides with integrated sequence of REDV-YGRKKRRQRRR-PKKKRKV-H_n_ (REDV-TAT-NLS-H_n_, n = 4, 8 and 12) were designed as the gene carrier for pZNF580 plasmid, and REDV-YGRKKRRQRRR-PKKKRKV-H_0_ without oligohistidine sequence was used as a control. In order to deliver genes into HUVECs selectively, REDV peptide was inserted into the peptide sequence due to its specific recognition of α4β1 integrin on the membrane of ECs. To increase the endosomal escape ability of the peptide gene carrier, oligohistidine H_n_ sequences were used to acquire cationic ions under acid condition especially in endosome/lysosome.

The REDV-TAT-NLS-H_n_/pZNF580 complexes were prepared by adding pZNF580 plasmid to REDV-TAT-NLS-H_n_ solution. The size of REDV-TAT-NLS-H_n_/pZNF580 complexes ranged from 117 to 288 nm and their zeta potential could be regulated by changing the weight ratio of peptide and pZNF580. According to the results of agarose gel electrophoresis, REDV-TAT-NLS-H_0_, REDV-TAT-NLS-H_4_, REDV-TAT-NLS-H_8_ and REDV-TAT-NLS-H_12_ peptides could completely condense the negative-charged pZNF580 plasmid to form gene complexes at the w/w ratio of 2, 2, 2 and 3, respectively. In addition, these peptides exhibited good blood compatibility and less destructed RBCs. The H residue possesses pH buffer capacity and hydrophobicity at pH 7.4. Thus, the positive charge of these peptides decreased when H_n_ sequence increased from 4 to 12. According to the cytotoxicity assay, the relative cell viability of the peptides and their gene complexes was higher than 80% even at a high concentration (120 μg mL^−1^), which showed a brilliant low cytotoxicity compared with PEI (25 kDa) [66]. The cellular uptake and MFI of the complexes increased when the H_n_ sequence length increased from 4 to 12. The reason is that the hydrophobicity of gene carriers expedites the hydrophobic interaction with cell membrane, which can promote their cellular internalization [58]. Moreover, the MFI of REDV-TAT-NLS-H_12_/Cy5-oligonucleotide complexes in HUVECs was about twofold as much as that in HUASMCs, which exhibited the specific recognition ability to HUVECs due to REDV in peptide sequence. Compared with the REDV-TAT-NLS-H_0_/pZNF580 complexes, REDV-TAT-NLS-H_n_/pZNF580 complexes showed higher gene delivery and transfection efficiency due to the synergistic effects of the peptide sequences to enhance cellular uptake, endosome/lysosome escape and nucleus translocation. The results of western blot analysis and PCR analysis also demonstrated the same tendency of ZNF580 expression at the protein level and mRNA level. For the HUVEC migration assay, the migration rate of REDV-TAT-NLS-H_n_/pZNF580 complexes groups was much higher than that of the REDV-TAT-NLS-H_0_/pZNF580 group because of their higher transfection efficiency. REDV-TAT-NLS-H_12_/pZNF580 group showed the highest migration rate (224 migrated cells), which was higher than the standard transfection reagent PEI 25 kDa/pZNF580 group [66]. Furthermore, according to the intercellular distribution assay, the REDV-TAT-NLS-H_12_/Cy5-oligonucleotide complexes showed the highest ability of endosome/lysosome escape.

In order to investigate the cellular uptake mechanism of the REDV-TAT-NLS-H_12_/pZNF580 complexes, cells were pre-treated with different inhibitors before transfection. The result demonstrated that clathrin-mediated endocytosis was the main pathway for the cellular uptake of REDV-TAT-NLS-H_12_/Cy5-oligonucleotide complexes, and other pathways including caveolae-mediated endocytosis, micropinocytosis and other endocytosis pathways also contributed to the cellular uptake. In addition, targeting REDV peptide in REDV-TAT-NLS-H_12_/Cy5-oligonucleotide complexes also benefited for the cellular uptake.

To evaluate the tube formation ability of HUVECs treated by REDV-TAT-NLS-H_n_/pZNF580 complexes, the angiogenesis assay was performed both in vitro and in vivo. The cells transfected with the REDV-TAT-NLS-H_n_/pZNF580 complexes could enhance the tube formation ability compared with the naked pZNF580 group. The angiogenesis ability of HUVECs transfected by REDV-TAT-NLS-H_12_/pZNF580 complexes was the highest among all of the groups. These complexes could efficiently deliver gene into cells.

According to the above results, the multifunctional REDV-TAT-NLS-H_n_ micelles could efficiently condense pZNF580, enhance gene delivery ability, and promote the migration, proliferation as well as neovascularization ability of HUVECs, which have great potential in the application of therapeutic vascularization for various vascular diseases.

## Conclusion

In summary, this paper designed the multifunctional targeting peptide sequences of REDV-YGRKKRRQRRR-PKKKRKV-H_n_ (n = 4, 8 and 12) as a gene carrier for improving the cellular uptake and gene delivery. TAT peptide and targeting REDV peptide benefited for the cellular uptake of pZNF580 plasmid in HUVECs. And the H_n_ sequence simultaneously improved the internalization efficiency and endosomal escape of REDV-TAT-NLS-H_n_/pZNF580 complexes, which further enhanced the expression of ZNF580 as well as the proliferation and migration ability of HUVECs. The expression of ZNF580 could enhance the tube formation ability of transfected HUVECs in vivo. Overall, these multifunctional peptide gene carriers provide an outstanding platform for rapid endothelialization and revascularization.

## Additional file

**Additional file 1: Figure S1.** Hydrodynamic diameter distribution of REDV-TAT-NLS-Hn micelles characterized by DLS. **Figure S2.** Hydrodynamic diameter distribution of REDV-TAT-NLS-H_n_/pZNF580 complexes (w/w = 1) characterized by DLS. **Figure S3.** Hydrodynamic diameter distribution of REDV-TAT-NLS-H_n_/pZNF580 complexes (w/w = 2) characterized by DLS. **Figure S4.** Hydrodynamic diameter distribution of REDV-TAT-NLS-H_n_/pZNF580 complexes (w/w = 3) characterized by DLS. **Figure S5.** Hydrodynamic diameter distribution of REDV-TAT-NLS-H_n_/pZNF580 complexes (w/w = 4) characterized by DLS. **Figure S6.** Hydrodynamic diameter distribution of REDV-TAT-NLS-H_n_/pZNF580 complexes (w/w = 5) characterized by DLS.
