# Supplementary material for: Oligohistidine and targeting peptide functionalized TAT-NLS for enhancing cellular uptake and promoting angiogenesis in vivo
Source: J Nanobiotechnology. 2018 Mar 26;16:29. doi: 10.1186/s12951-018-0358-x (PMC5870920; doi:10.1186/s12951-018-0358-x)
Supplement: Supplementary file 1 — Additional file 1: Figure S1. Hydrodynamic diameter distribution of REDV-TAT-NLS-Hn micelles characterized by DLS. Figure S2. Hydrodynamic diameter distribution of REDV-TAT-NLS-Hn/pZNF580 complexes (w/w = 1) characterized by DLS. Figure S3. Hydrodynamic diameter distribution of REDV-TAT-NLS-Hn/pZNF580 complexes (w/w = 2) characterized by DLS. Figure S4. Hydrodynamic diameter distribution of REDV-TAT-NLS-Hn/pZNF580 complexes (w/w = 3) characterized by DLS. Figure S5. Hydrodynamic diameter distribution of REDV-TAT-NLS-Hn/pZNF580 complexes (w/w = 4) characterized by DLS. Figure S6. Hydrodynamic diameter distribution of REDV-TAT-NLS-Hn/pZNF580 complexes (w/w = 5) characterized by DLS. [file 12951_2018_358_MOESM1_ESM.doc]

**Electronic Supplementary Information**

**Oligo****histidine and targeting peptide functionalized TAT-NLS for enhancing cellular uptake and promoting angiogenesis in vivo**

Qian Li^1^, Xuefang Hao^1^, Syed Saqib Ali Zaidi^1^, Jintang Guo^1,2^, Xiangkui Ren*^1,2^, Yakai Feng*^1,2,3^, Changcan Shi^*4,5^, Wencheng Zhang^6^

Qian Li, School of Chemical Engineering and Technology, Tianjin University, Tianjin 300350, China. Email: [liqian200512@126.com](mailto:liqian200512@126.com)

Xuefang Hao, School of Chemical Engineering and Technology, Tianjin University, Tianjin 300350, China. Email: [hxf15175374404@126.com](mailto:hxf15175374404@126.com)

Syed Saqib Ali Zaidi，School of Chemical Engineering and Technology, Tianjin University, Tianjin 300350, China. Email: saqib_48@outlook.com

Jintang Guo, School of Chemical Engineering and Technology, Tianjin University, Tianjin 300350, China. Email: [jtguo@tju.edu.cn](mailto:jtguo@tju.edu.cn)

Xiangkui Ren, School of Chemical Engineering and Technology, Tianjin University, Tianjin 300350, China. Email: [renxiangkui@tju.edu.cn](mailto:renxiangkui@tju.edu.cn)

Changcan Shi, Wenzhou Institute of Biomaterials and Engineering, CNITECH, CAS, Wenzhou, Zhejiang 325011, China. Email: [shichangcan@126.com](mailto:shichangcan@126.com)

Wencheng Zhang, Department of Physiology and Pathophysiology, Logistics University of Chinese People’s Armed Police Force, Tianjin 300309, China. [wenchen78084@163.com](mailto:wenchen78084@163.com)

Corresponding Author: Y. Feng, School of Chemical Engineering and Technology, Tianjin University, Tianjin 300350, China. Email: [yakaifeng@tju.edu.cn](mailto:yakaifeng@tju.edu.cn) (Y. Feng)

The file includes:

**Fig. S1**: Hydrodynamic diameter distribution of of REDV-TAT-NLS-H_n_ micelles characterized by DLS. A: REDV-TAT-NLS-H_0_ micelles, B: REDV-TAT-NLS-H_4_ micelles, C: REDV-TAT-NLS-H_8_ micelles, D: REDV-TAT-NLS-H_12_ micelles.

**Fig. S2** Hydrodynamic diameter distribution of REDV-TAT-NLS-H_n_/pZNF580 complexes (w/w = 1) characterized by DLS. A: REDV-TAT-NLS-H_0_/pZNF580 complexes (w/w = 1), B: REDV-TAT-NLS-H_4_/pZNF580 complexes (w/w = 1), C: REDV-TAT-NLS-H_8_/pZNF580 complexes (w/w = 1), D: REDV-TAT-NLS-H_12_/pZNF580 complexes (w/w = 1).

**Fig. S3** Hydrodynamic diameter distribution of REDV-TAT-NLS-H_n_/pZNF580 complexes (w/w = 2) characterized by DLS. A: REDV-TAT-NLS-H_0_/pZNF580 complexes (w/w = 2), B: REDV-TAT-NLS-H_4_/pZNF580 complexes (w/w = 2), C: REDV-TAT-NLS-H_8_/pZNF580 complexes (w/w = 2), D: REDV-TAT-NLS-H_12_/pZNF580 complexes (w/w = 2).

**Fig. S4** Hydrodynamic diameter distribution of REDV-TAT-NLS-H_n_/pZNF580 complexes (w/w = 3) characterized by DLS. A: REDV-TAT-NLS-H_0_/pZNF580 complexes (w/w = 3), B: REDV-TAT-NLS-H_4_/pZNF580 complexes (w/w = 3), C: REDV-TAT-NLS-H_8_/pZNF580 complexes (w/w = 3), D: REDV-TAT-NLS-H_12_/pZNF580 complexes (w/w = 3).

**Fig. S5** Hydrodynamic diameter distribution of REDV-TAT-NLS-H_n_/pZNF580 complexes (w/w = 4) characterized by DLS. A: REDV-TAT-NLS-H_0_/pZNF580 complexes (w/w = 4), B: REDV-TAT-NLS-H_4_/pZNF580 complexes (w/w = 4), C: REDV-TAT-NLS-H_8_/pZNF580 complexes (w/w = 4), D: REDV-TAT-NLS-H_12_/pZNF580 complexes (w/w = 4).

**Fig. S6** Hydrodynamic diameter distribution of REDV-TAT-NLS-H_n_/pZNF580 complexes (w/w = 5) characterized by DLS. A: REDV-TAT-NLS-H_0_/pZNF580 complexes (w/w = 5), B: REDV-TAT-NLS-H_4_/pZNF580 complexes (w/w = 5), C: REDV-TAT-NLS-H_8_/pZNF580 complexes (w/w = 5), D: REDV-TAT-NLS-H_12_/pZNF580 complexes (w/w = 5).


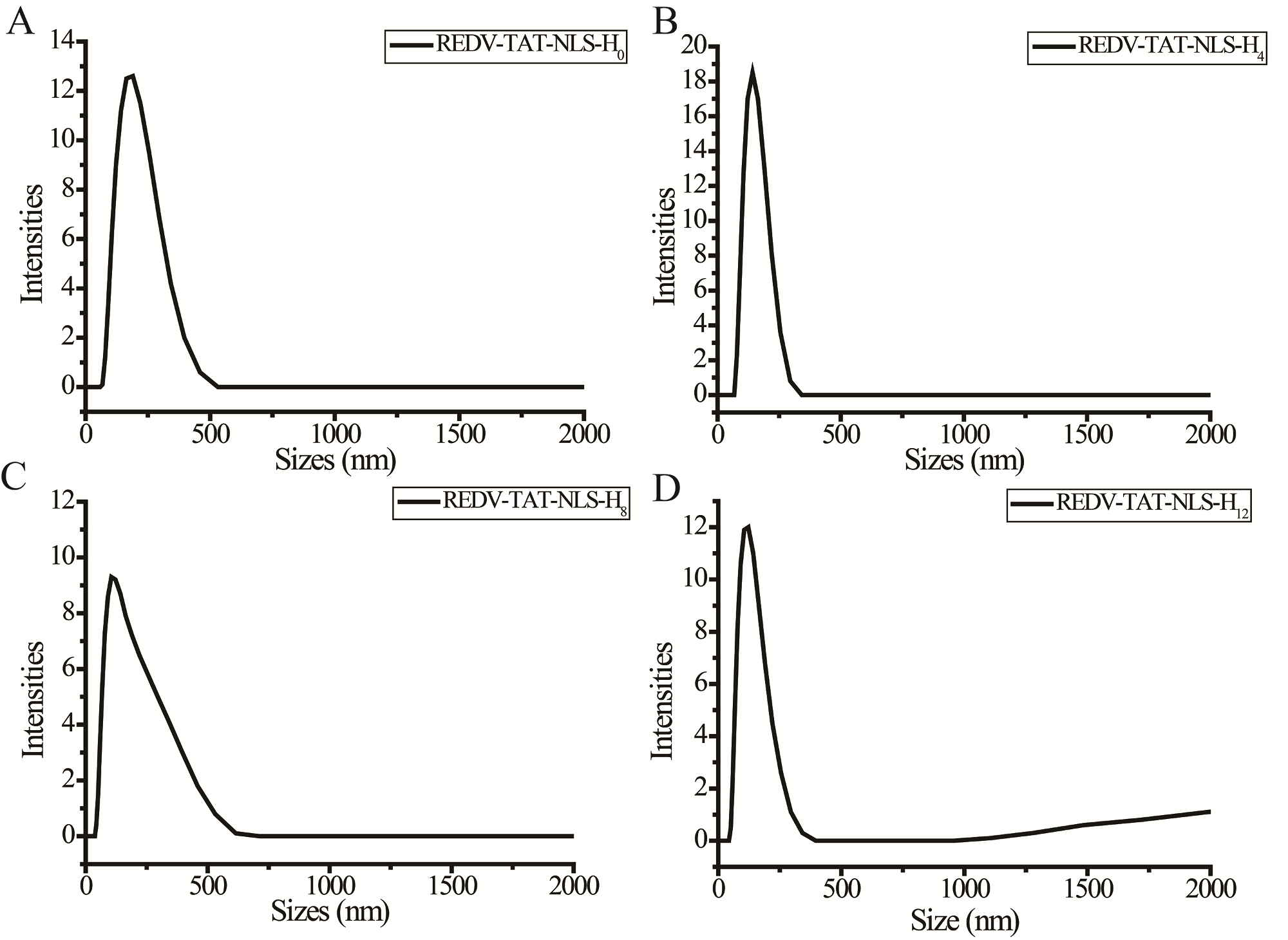


**Fig. S1** Hydrodynamic diameter distribution of REDV-TAT-NLS-H_n_ micelles characterized by DLS.


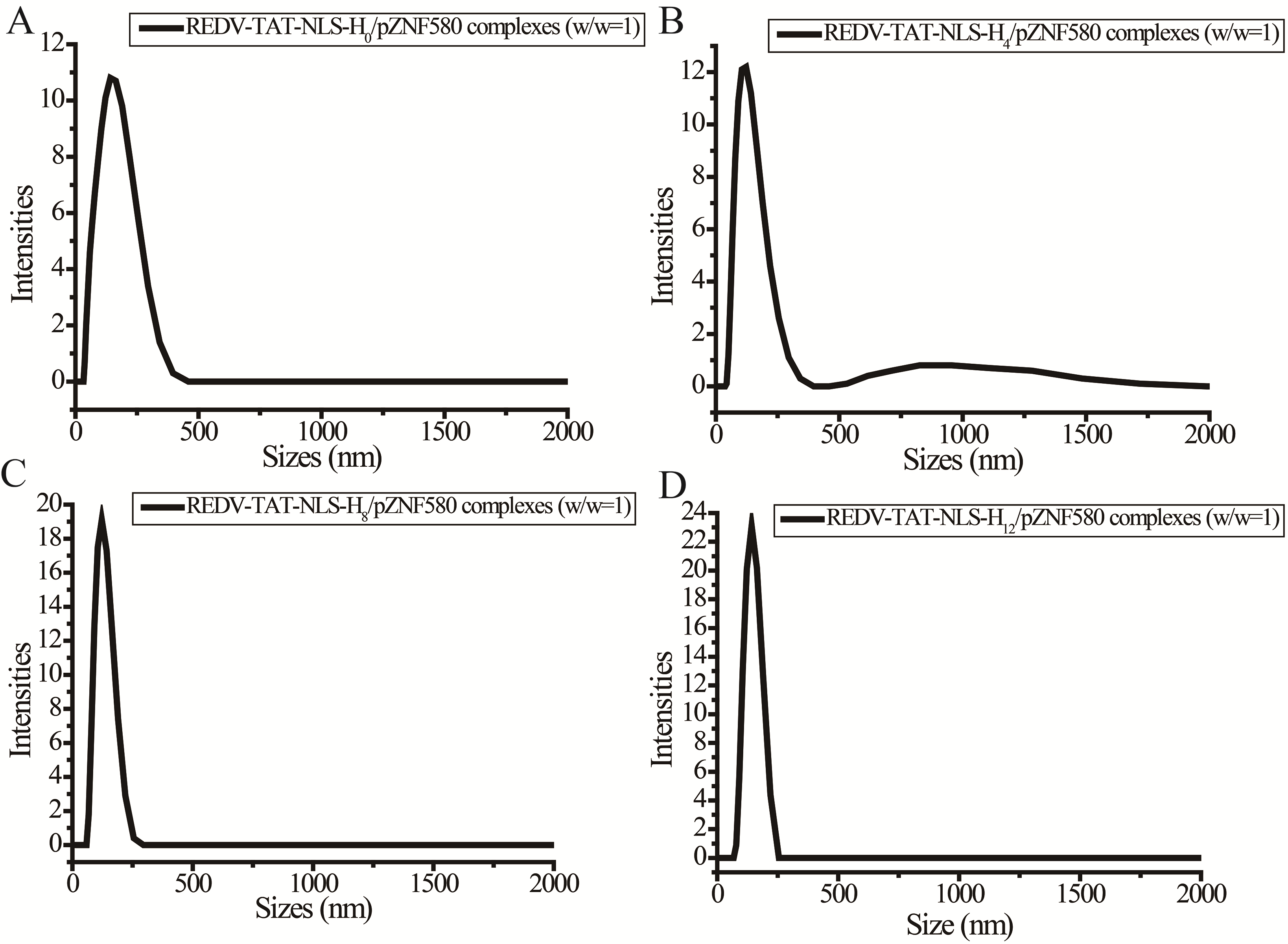


**Fig. S2** Hydrodynamic diameter distribution of REDV-TAT-NLS-H_n_/pZNF580 complexes (w/w = 1) characterized by DLS.


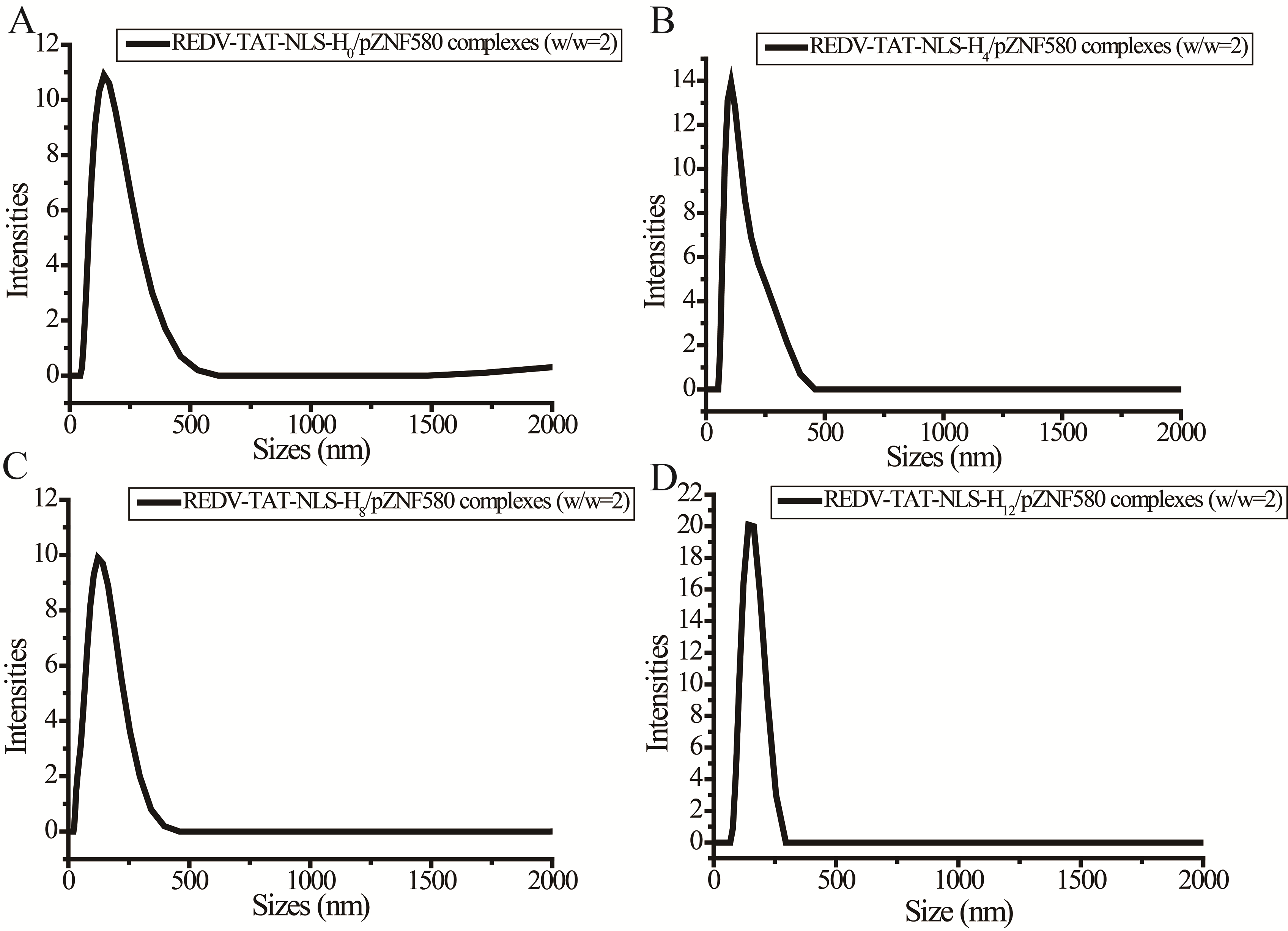


**Fig. S3** Hydrodynamic diameter distribution of REDV-TAT-NLS-H_n_/pZNF580 complexes (w/w=2) characterized by DLS.


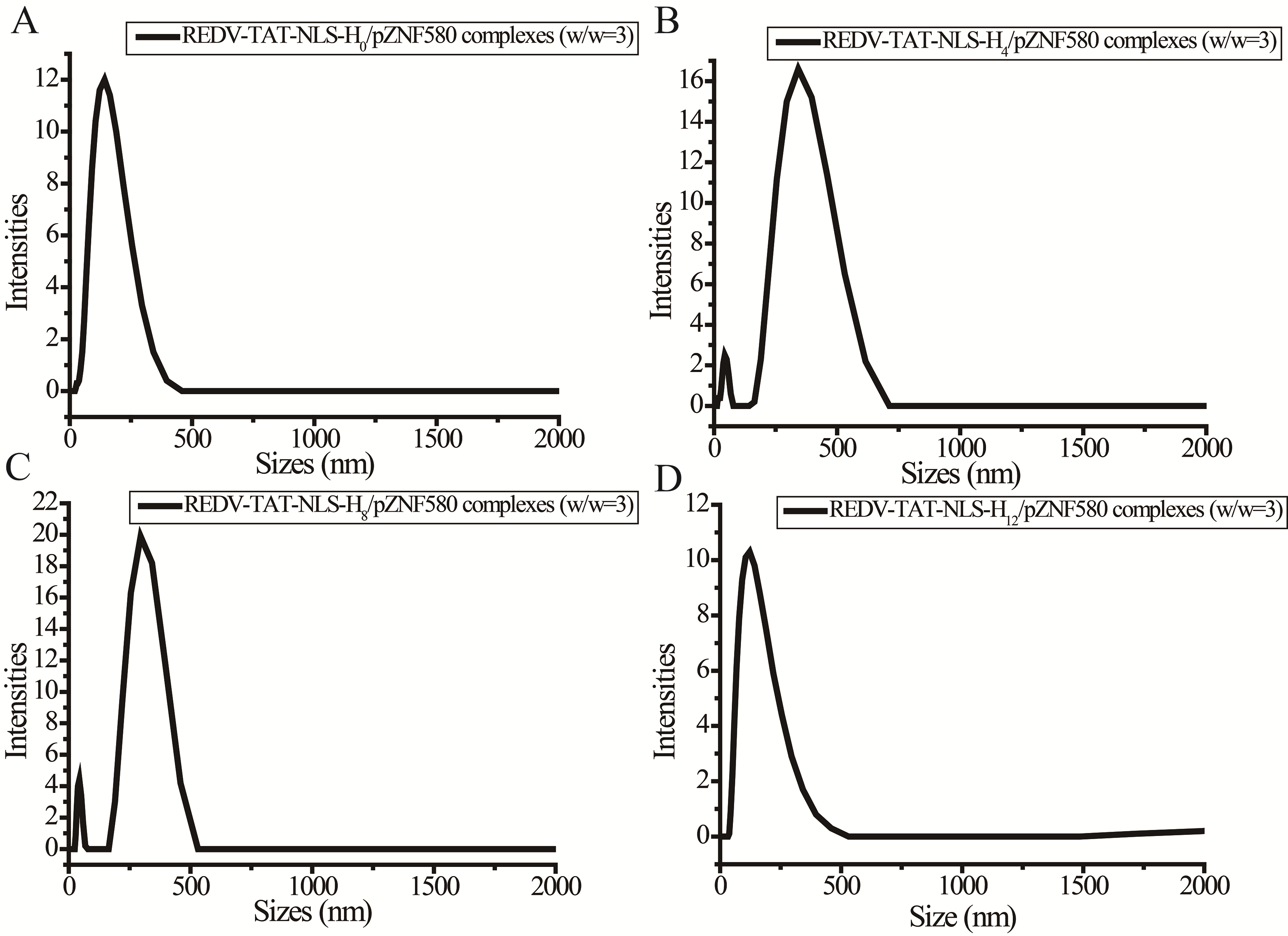


**Fig. S4** Hydrodynamic diameter distribution of REDV-TAT-NLS-H_n_/pZNF580 complexes (w/w=3) characterized by DLS.


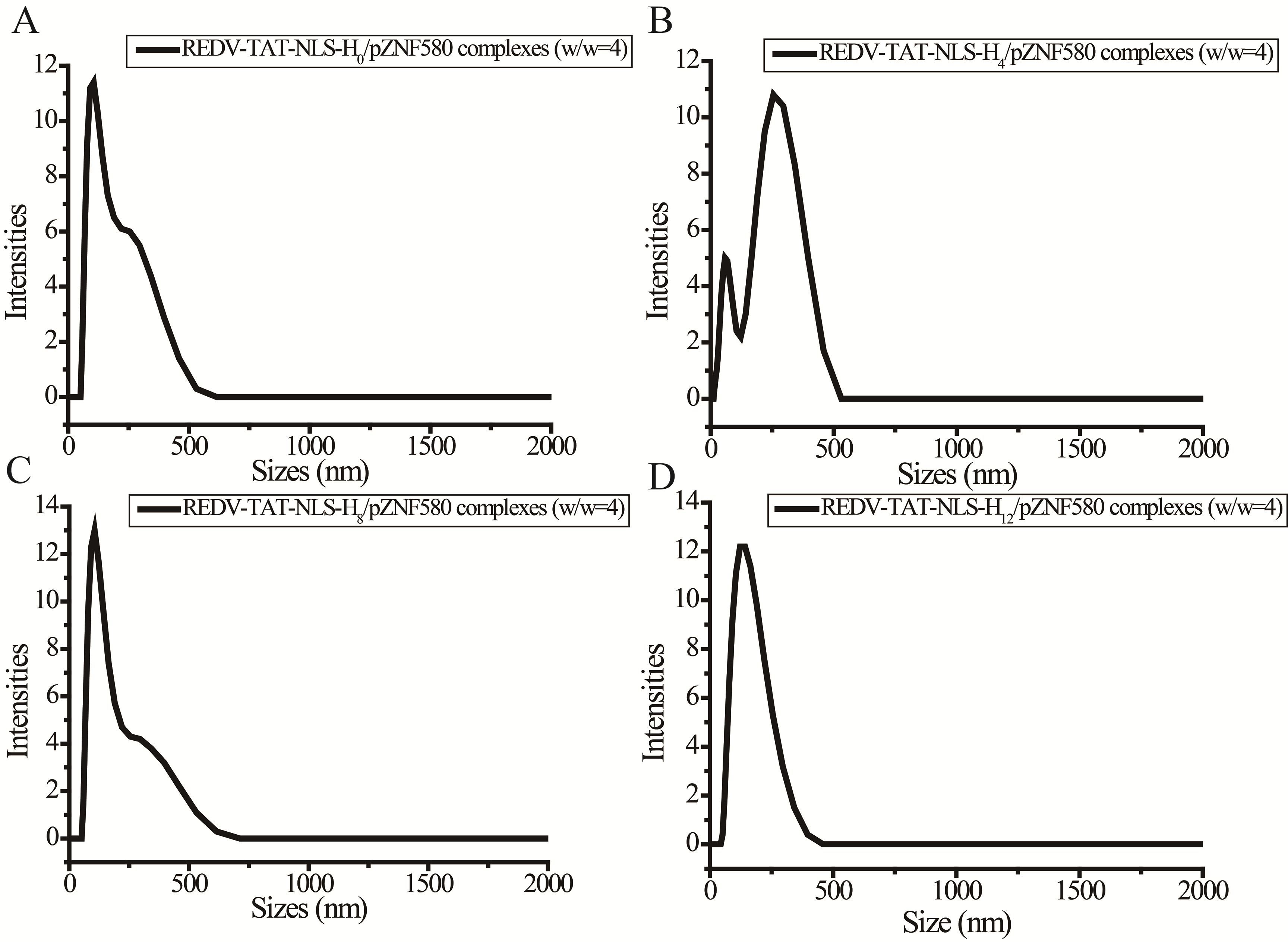


**Fig. S5** Hydrodynamic diameter distribution of REDV-TAT-NLS-H_n_/pZNF580 complexes (w/w=4) characterized by DLS.


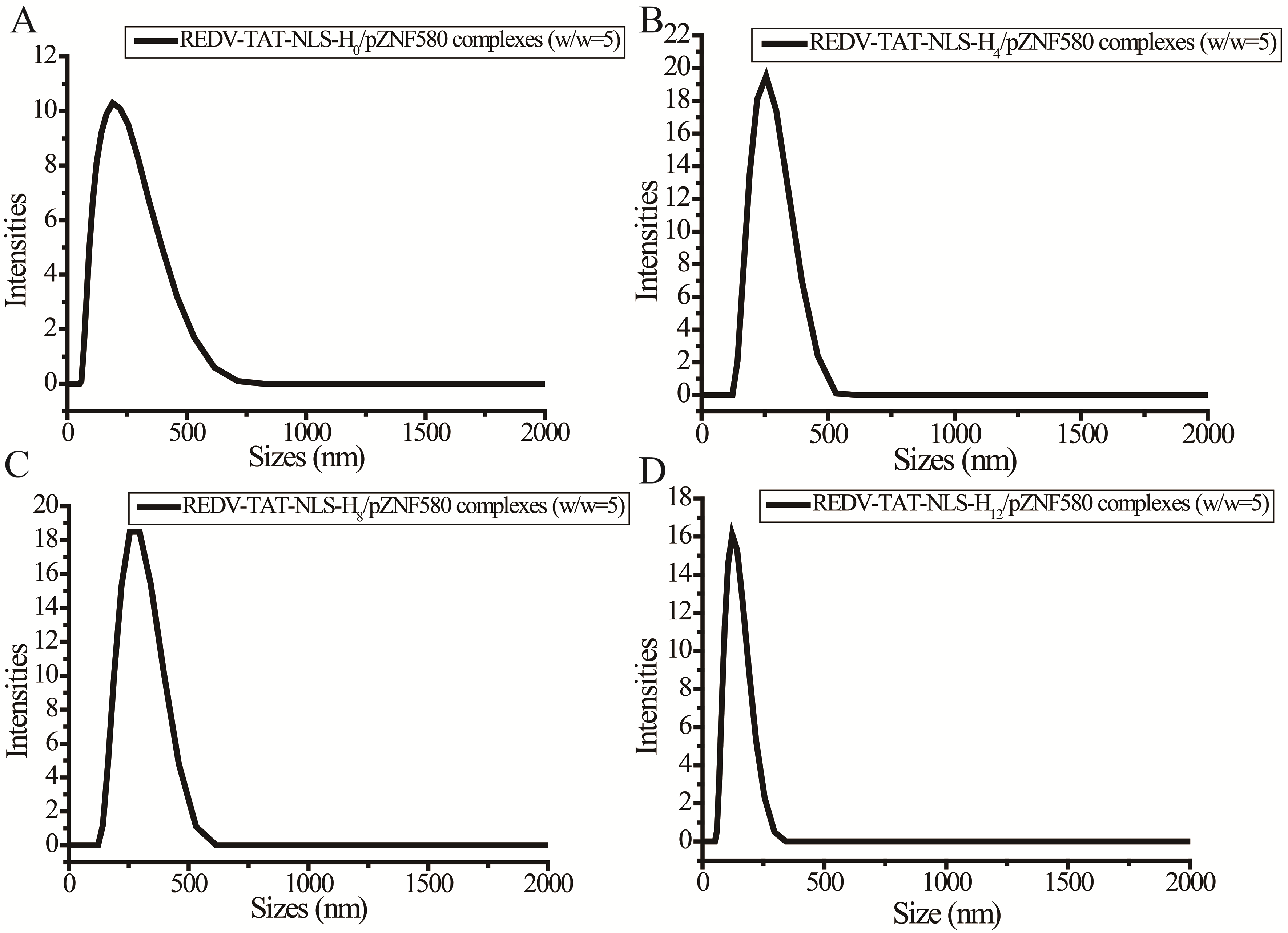


**Fig. S6** Hydrodynamic diameter distribution of REDV-TAT-NLS-H_n_/pZNF580 complexes (w/w=5) characterized by DLS.
